# Supplementary material for: Real-world effects of alcohol on heart rate, sleep, and physical activity by age and sex
Source: PLOS Digit Health. 2026 Mar 9;5(3):e0001284. doi: 10.1371/journal.pdig.0001284 (PMC12970902; doi:10.1371/journal.pdig.0001284)
Supplement: S1 Table — (DOCX) [file pdig.0001284.s001.docx]

| **Supplemental Table 1.**  Estimated alcohol drinking frequency differences in physiological and behavioral outcomes by number of drinks (within-person centered) | | | |
| --- | --- | --- | --- |
| **Number of Drinks**  **(within-person centered)** | **Low - High Estimate (99.9% CI)** | **Effect Size (ES)** | **P-Value** |
| **Resting Heart Rate (bpm)** | | | |
| -1 | 0.01 (–0.54, 0.56) | <0.01 | P = .958 |
| 1 | -0.02 (-0.08, 0.04) | <0.01 | P = .227 |
| 3 | -0.23 (-0.33, -0.14) | 0.05 | P < .001 |
| 5 | -0.15 (-0.29, 0.00) | 0.03 | P = .001 |
| **Heart Rate Variability (ms)** | | | |
| -1 | 6.74 (3.78, 9.70) | 0.53 | P < .001 |
| 1 | -0.05 (-0.23, 0.12) | <0.01 | P = .322 |
| 3 | -0.31 (-0.58, -0.05) | 0.03 | P = .001 |
| 5 | -1.77 (-2.20, -1.34) | 0.14 | P < .001 |
| **Sleep Duration (min)** | | | |
| -1 | 15.40 (9.78, 21.02) | 0.23 | P < .001 |
| 1 | 2.82 (1.96, 3.68) | 0.04 | P < .001 |
| 3 | -1.77 (-3.08, -0.45) | 0.03 | P < .001 |
| 5 | -3.62 (−5.71, -1.53) | 0.05 | P < .001 |
| **Activity Load (AU)** | | | |
| -1 | 3.19 (–2.78, 9.18) | 0.03 | P = .078 |
| 1 | –4.02 (-5.38, –2.67) | 0.04 | P < .001 |
| 3 | –4.53 (–6.53, –2.53) | 0.04 | P < .001 |
| 5 | –6.35 (–9.42, -3.28) | 0.06 | P < .001 |
| Estimates reflect Low - High frequency drinker contrasts derived from estimate marginal means using generalized additive models, with corresponding 99.9% confidence intervals. ES = standardized effect size. These results correspond to the modeled associations shown in **S1** **Fig**. | | | |
