# Supplementary material for: Real-world effects of alcohol on heart rate, sleep, and physical activity by age and sex
Source: PLOS Digit Health. 2026 Mar 9;5(3):e0001284. doi: 10.1371/journal.pdig.0001284 (PMC12970902; doi:10.1371/journal.pdig.0001284)
Supplement: S2 Table — (DOCX) [file pdig.0001284.s002.docx]

| **Supplemental Table 2.** Estimated differences in physiological and behavioral outcomes by number of drinks (within-person centered) by biological sex | | |
| --- | --- | --- |
| **Number of Drinks (within-person centered)** | **Female** | **Male** |
| **Resting Heart Rate (bpm)** | | |
| 1 vs –1 | 2.76 (2.65, 2.87); ES = 0.61; P < .001 | 2.35 (2.28, 2.42); ES = 0.52; P < .001 |
| 3 vs 1 | 3.11 (3.04, 3.19); ES = 0.69; P < .001 | 2.78 (2.72, 2.84); ES = 0.61; P < .001 |
| 5 vs 3 | 2.65 (2.53, 2.77); ES = 0.58; P < .001 | 2.53 (2.44, 2.61); ES = 0.56; P < .001 |
| **Heart Rate Variability (ms)** | | |
| 1 vs –1 | –3.75 (–4.05, –3.45); ES = 0.30; P < .001 | –3.27 (–3.47, –3.08); ES = 0.26; P < .001 |
| 3 vs 1 | –5.25 (–5.45, –5.04); ES = 0.42; P < .001 | –4.54 (–4.70, –4.38); ES = 0.36; P < .001 |
| 5 vs 3 | –5.58 (–5.89, –5.26); ES = 0.45; P < .001 | –5.12 (–5.34, –4.89); ES = 0.41; P < .001 |
| **Sleep Duration (min)** | | |
| 1 vs –1 | –8.37 (–10.01, –6.74); ES = 0.12; P < .001 | –6.94 (–7.99, –5.89); ES = 0.10; P < .001 |
| 3 vs 1 | –16.62 (–17.72, –15.52); ES = 0.24; P < .001 | –13.28 (–14.14, –12.42); ES = 0.19; P < .001 |
| 5 vs 3 | –19.40 (–21.09, –17.71); ES = 0.28; P < .001 | –15.71 (–16.87, –14.55); ES = 0.23; P < .001 |
| **Activity Load (AU)** | | |
| 1 vs –1 | –3.92 (–6.51, –1.34); ES = 0.04; P < .001 | –2.42 (–4.06, –0.78); ES = 0.02; P < .001 |
| 3 vs 1 | –13.28 (–14.98, –11.58); ES = 0.12; P < .001 | –8.84 (–10.16, –7.51); ES = 0.08; P < .001 |
| 5 vs 3 | –13.60 (–16.15, –11.05); ES = 0.13; P < .001 | –9.48 (–11.23, –7.74); ES = 0.09; P < .001 |
| Estimates reflect dose-response contrasts between drink number and physiological or behavioral responses, with corresponding 99.9% confidence intervals, stratified by biological sex. ES = standardized effect size. These results correspond to the modeled associations shown in **Fig 2A-D**. | | |
