# Supplementary material for: Real-world effects of alcohol on heart rate, sleep, and physical activity by age and sex
Source: PLOS Digit Health. 2026 Mar 9;5(3):e0001284. doi: 10.1371/journal.pdig.0001284 (PMC12970902; doi:10.1371/journal.pdig.0001284)
Supplement: S3 Table — (DOCX) [file pdig.0001284.s003.docx]

| **Supplemental Table 3.**  Estimated biological sex differences in physiological and behavioral outcomes by number of drinks (within-person centered) | | | |
| --- | --- | --- | --- |
| **Number of Drinks (within-person centered)** | **Female – Male Estimate (99.9% CI)** | **Effect Size (ES)** | **P-Value** |
| **Resting Heart Rate (bpm)** | | | |
| –1 | −0.21 (−0.31, −0.11) | 0.05 | <.001 |
| 1 | 0.20 (0.15, 0.25) | 0.04 | <.001 |
| 3 | 0.54 (0.46, 0.62) | 0.12 | <.001 |
| 5 | 0.67 (0.53, 0.80) | 0.15 | <.001 |
| **Heart Rate Variability (ms)** | | | |
| –1 | −0.13 (−0.40, 0.15) | 0.01 | .131 |
| 1 | −0.61 (−0.73, −0.48) | 0.05 | <.001 |
| 3 | −1.31 (−1.52, −1.10) | 0.11 | <.001 |
| 5 | −1.77 (−2.14, −1.41) | 0.14 | <.001 |
| **Sleep Duration (min)** | | | |
| –1 | 3.41 (1.92, 4.89) | 0.05 | <.001 |
| 1 | 1.98 (1.31, 2.64) | 0.03 | <.001 |
| 3 | −1.36 (−2.49, −0.22) | 0.02 | <.001 |
| 5 | −5.05 (−7.02, −3.08) | 0.07 | <.001 |
| **Activity Load (AU)** | | | |
| –1 | −0.25 (−2.60, 2.10) | <0.01 | .725 |
| 1 | −1.75 (−2.79, −0.71) | 0.02 | <.001 |
| 3 | −6.19 (−7.96, −4.42) | 0.06 | <.001 |
| 5 | −10.31 (−13.36, −7.25) | 0.10 | <.001 |
| Estimates reflect Female – Male contrasts at different drink quantities derived from estimate marginal means using generalized additive models, with corresponding 99.9% confidence intervals. ES = standardized effect size. These results correspond to the modeled associations shown in **Fig 2A-D**. | | | |
