# Supplementary material for: Real-world effects of alcohol on heart rate, sleep, and physical activity by age and sex
Source: PLOS Digit Health. 2026 Mar 9;5(3):e0001284. doi: 10.1371/journal.pdig.0001284 (PMC12970902; doi:10.1371/journal.pdig.0001284)
Supplement: S4 Table — (DOCX) [file pdig.0001284.s004.docx]

| **Supplemental Table 4.** Estimated biological sex differences in physiological and behavioral outcomes by number of drinks | | | |
| --- | --- | --- | --- |
| **Number of Drinks** | **Female – Male Estimate (99.9% CI)** | **Effect Size (ES)** | **P-Value** |
| **Resting Heart Rate (bpm)** | | | |
| 1 | 0.04 (−0.01, 0.10) | 0.01 | .015 |
| 3 | 0.33 (0.27, 0.39) | 0.07 | <.001 |
| 5 | 0.57 (0.47, 0.67) | 0.12 | <.001 |
| 7 | 0.42 (0.24, 0.59) | 0.09 | <.001 |
| **Heart Rate Variability (ms)** | | | |
| 1 | −0.32 (−0.47, −0.17) | 0.03 | <.001 |
| 3 | −0.82 (−0.98, −0.67) | 0.07 | <.001 |
| 5 | −1.44 (−1.70, −1.17) | 0.11 | <.001 |
| 7 | −1.28 (−1.75, −0.81) | 0.10 | <.001 |
| **Sleep Duration (hrs)** | | | |
| 1 | 0.04 (0.03, 0.05) | 0.03 | <.001 |
| 3 | 0.03 (0.02, 0.04) | 0.03 | <.001 |
| 5 | −0.04 (−0.06, −0.01) | 0.03 | <.001 |
| 7 | −0.07 (−0.11, −0.03) | 0.06 | <.001 |
| **Activity Load (AU)** | | | |
| 1 | −0.47 (−1.74, 0.81) | <0.01 | .228 |
| 3 | −3.02 (−4.29, −1.74) | 0.03 | <.001 |
| 5 | −7.37 (−9.50, −5.23) | 0.07 | <.001 |
| 7 | −9.10 (−12.61, −5.60) | 0.08 | <.001 |
| Estimates reflect Female – Male contrasts at different drink quantities derived from estimate marginal means using generalized additive models, with corresponding 99.9% confidence intervals. ES = standardized effect size. These results correspond to the modeled associations shown in **S2** **Fig**. | | | |
