# Supplementary material for: Real-world effects of alcohol on heart rate, sleep, and physical activity by age and sex
Source: PLOS Digit Health. 2026 Mar 9;5(3):e0001284. doi: 10.1371/journal.pdig.0001284 (PMC12970902; doi:10.1371/journal.pdig.0001284)
Supplement: S5 Table — (DOCX) [file pdig.0001284.s005.docx]

| **Supplemental Table 5.** Estimated differences in physiological and behavioral outcomes by time between last alcoholic drink and bedtime (within-person centered) by biological sex | | |
| --- | --- | --- |
| **Time from Last Drink to Bed (within-person centered)** | **Female** | **Male** |
| **Resting Heart Rate (bpm)** | | |
| –60 vs –180 min | –0.69 (–0.82, –0.55); ES=0.15; P<.001 | –0.48 (–0.60, –0.36); ES=0.11; P<.001 |
| 60 vs –60 min | –0.87 (–0.94, –0.80); ES=0.19; P<.001 | –0.70 (–0.76, –0.64); ES=0.15; P<.001 |
| 180 vs 60 min | –0.61 (–0.72, –0.50); ES=0.13; P<.001 | –0.48 (–0.58, –0.39); ES=0.11; P<.001 |
| 300 vs 180 min | –0.48 (–0.63, –0.32); ES=0.10; P<.001 | –0.40 (–0.55, –0.26); ES=0.09; P<.001 |
| 420 vs 300 min | –0.15 (–0.40, 0.10); ES=0.03; P=.108 | –0.11 (–0.34, 0.11); ES=0.02; P=.261 |
| **Heart Rate Variability (ms)** | | |
| –60 vs –180 min | 1.27 (0.92, 1.62); ES=0.10; P<.001 | 0.96 (0.68, 1.24); ES=0.08; P<.001 |
| 60 vs –60 min | 1.50 (1.30, 1.69); ES=0.12; P<.001 | 1.30 (1.15, 1.46); ES=0.10; P<.001 |
| 180 vs 60 min | 1.03 (0.75, 1.31); ES=0.08; P<.001 | 0.99 (0.77, 1.21); ES=0.08; P<.001 |
| 300 vs 180 min | 1.19 (0.78, 1.59); ES=0.09; P<.001 | 0.94 (0.63, 1.25); ES=0.08; P<.001 |
| 420 vs 300 min | 1.19 (0.55, 1.82); ES=0.09; P<.001 | 0.89 (0.42, 1.36); ES=0.07; P<.001 |
| **Sleep Duration (min)** | | |
| –60 vs –180 min | -10.64 (-12.85, -8.42); ES=0.16; P<.001 | -11.40 (-13.25, -9.54); ES=0.17; P<.001 |
| 60 vs –60 min | -18.86 (-20.02, -17.69); ES=0.28; P<.001 | -19.70 (-20.66, -18.74); ES=0.29; P<.001 |
| 180 vs 60 min | -8.73 (-10.54, -6.91); ES=0.13; P<.001 | -9.66 (-11.19, -8.14); ES=0.14; P<.001 |
| 300 vs 180 min | -1.77 (-4.59, 1.04); ES=0.03; P=.084 | -5.10 (-7.50, -2.71); ES=0.07; P<.001 |
| 420 vs 300 min | -29.64 (-34.45, -24.84); ES=0.43; P<.001 | -28.25 (-32.17, -24.33); ES=0.41; P<.001 |
| **Activity Load (AU)** | | |
| –60 vs –180 min | 3.94 (0.83, 7.06); ES=0.04; P<.001 | 1.80 (–0.57, 4.17); ES=0.02; P=.023 |
| 60 vs –60 min | –0.49 (–2.19, 1.21); ES<0.01; P=.788 | –0.57 (–1.88, 0.75); ES=0.01; P=.422 |
| 180 vs 60 min | –2.49 (–4.97, –0.02); ES=0.02; P<.001 | –1.51 (–3.34, 0.32); ES=0.01; P=.011 |
| 300 vs 180 min | 2.08 (–1.50, 5.67); ES=0.02; P=.139 | –1.53 (–4.08, 1.03); ES=0.01; P=.120 |
| 420 vs 300 min | –3.52 (–9.14, 2.10); ES=0.03; P=.093 | –4.10 (–8.00, –0.21); ES=0.04; P<.001 |
| Estimates reflect dose-response contrasts between drink timing and physiological or behavioral responses, with corresponding 99.9% confidence intervals, stratified by sex. ES = standardized effect size. These results correspond to the modeled associations shown in **Fig 3A-D**. | | |
