# Supplementary material for: Real-world effects of alcohol on heart rate, sleep, and physical activity by age and sex
Source: PLOS Digit Health. 2026 Mar 9;5(3):e0001284. doi: 10.1371/journal.pdig.0001284 (PMC12970902; doi:10.1371/journal.pdig.0001284)
Supplement: S6 Table — (DOCX) [file pdig.0001284.s006.docx]

| **Supplemental Table 6.**  Estimated biological sex differences in physiological and behavioral outcomes at varying times from last alcoholic drink to bedtime (within-person centered) | | | |
| --- | --- | --- | --- |
| **Time from Last Drink to Bed (within-person centered)** | **Female – Male Estimate (99.9% CI)** | **Effect Size (ES)** | **P-Value** |
| **Resting Heart Rate (bpm)** | | | |
| –180 min | 0.47 (0.33, 0.62) | 0.10 | < .001 |
| –60 min | 0.27 (0.22, 0.32) | 0.06 | < .001 |
| 60 min | 0.10 (0.03, 0.16) | 0.02 | < .001 |
| 180 min | -0.03 (–0.14, 0.09) | 0.01 | .454 |
| 300 min | –0.11 (–0.28, 0.07) | 0.02 | .054 |
| 420 min | –0.14 (–0.41, 0.14) | 0.03 | .098 |
| **Heart Rate Variability (ms)** | | | |
| -180 min | -1.05 (-1.41, -0.70) | 0.08 | <.001 |
| -60 min | -0.75 (-0.88, -0.61) | 0.06 | < .001 |
| 60 min | -0.55 (-0.72, -0.39) | 0.04 | < .001 |
| 180 min | -0.51 (-0.81, -0.22) | 0.04 | < .001 |
| 300 min | -0.27 (-0.71, 0.17) | 0.02 | .045 |
| 420 min | 0.02 (-0.68, 0.72) | <0.01 | .919 |
| **Sleep Duration (min)** | | | |
| –180 min | 0.01 (-2.22, 2.24) | <0.01 | .986 |
| –60 min | 0.77 (-0.02, 1.56) | 0.01 | .001 |
| 60 min | 1.61 (0.64, 2.58) | 0.02 | < .001 |
| 180 min | 2.55 (0.70, 4.40) | 0.04 | < .001 |
| 300 min | 5.88 (3.04, 8.72) | 0.09 | < .001 |
| 420 min | 4.49 (–0.30, 9.27) | 0.07 | .002 |
| **Activity Load (AU)** | | | |
| –180 min | -4.77 (-7.84, -1.58) | 0.04 | < .001 |
| –60 min | -2.57 (-3.73, -1.41) | 0.02 | < .001 |
| 60 min | -2.50 (-3.93, -1.06) | 0.02 | < .001 |
| 180 min | -3.48 (-6.07, -0.89) | 0.03 | < .001 |
| 300 min | 0.13 (-3.72, 3.99) | <0.01 | .909 |
| 420 min | 0.72 (-5.35, 6.79) | 0.01 | .697 |
| Estimates reflect Female – Male contrasts at different drink timings derived from estimate marginal means using generalized additive models, with corresponding 99.9% confidence intervals. ES = standardized effect size. These results correspond to the modeled associations shown in **Fig 3A-D**. | | | |
