# Supplementary material for: Real-world effects of alcohol on heart rate, sleep, and physical activity by age and sex
Source: PLOS Digit Health. 2026 Mar 9;5(3):e0001284. doi: 10.1371/journal.pdig.0001284 (PMC12970902; doi:10.1371/journal.pdig.0001284)
Supplement: S7 Table — (DOCX) [file pdig.0001284.s007.docx]

| **Supplemental Table 7.** Estimated differences in physiological and behavioral outcomes by number of drinks (within-person centered) by age group | | | | | |
| --- | --- | --- | --- | --- | --- |
| **Number of Drinks (within-person centered)** | **20–29 yrs** | **30–39 yrs** | **40–49 yrs** | **50–59 yrs** | **60+ yrs** |
| **Resting Heart Rate (bpm)** | | | | | |
| **1 vs –1** | **2.58 (2.41, 2.75);**  **ES=0.57; P < .001** | **2.69 (2.55, 2.83);**  **ES=0.59; P < .001** | **2.56 (2.44, 2.68);**  **ES=0.56; P < .001** | **2.42 (2.31, 2.53);**  **ES=0.53; P < .001** | **2.23 (2.11, 2.35);**  **ES=0.49; P < .001** |
| **3 vs 1** | **3.27 (3.15, 3.38);**  **ES=0.72; P < .001** | **3.13 (3.02, 3.23);**  **ES=0.69; P < .001** | **2.95 (2.85, 3.04);**  **ES=0.65; P < .001** | **2.69 (2.59, 2.78);**  **ES=0.59; P < .001** | **2.47 (2.34, 2.59);**  **ES=0.54; P < .001** |
| **5 vs 3** | **2.92 (2.78, 3.05);**  **ES=0.64; P < .001** | **2.78 (2.64, 2.92);**  **ES=0.61; P < .001** | **2.48 (2.34, 2.62);**  **ES=0.55; P < .001** | **1.93 (1.77, 2.08);**  **ES=0.42; P < .001** | **1.58 (1.34, 1.82);**  **ES=0.35; P < .001** |
| **Heart Rate Variability (ms)** | | | | | |
| 1 vs –1 | –5.70 (–6.16, –5.23); ES=0.46; P<.001 | –4.99 (–5.38, –4.60); ES=0.40; P<.001 | –3.60 (–3.94, –3.26); ES=0.29; P<.001 | –2.54 (–2.83, –2.25); ES=0.20; P<.001 | –2.56 (–2.88, –2.24); ES=0.21; P<.001 |
| 3 vs 1 | –7.93 (–8.23, –7.64); ES=0.64; P<.001 | –6.24 (–6.52, –5.96); ES=0.50; P<.001 | –4.36 (–4.63, –4.10); ES=0.35; P<.001 | –3.10 (–3.35, –2.85); ES=0.25; P<.001 | –2.70 (–2.99, –2.40); ES=0.22; P<.001 |
| 5 vs 3 | –7.42 (–7.79, –7.06); ES=0.60; P<.001 | –5.54 (–5.92, –5.16); ES=0.45; P<.001 | –3.70 (–4.09, –3.30); ES=0.30; P<.001 | –2.40 (–2.80, –2.01); ES=0.19; P<.001 | –2.33 (–2.86, –1.80); ES=0.19; P<.001 |
| **Sleep Duration (min)** | | | | | |
| **1 vs –1** | –12.44 (–14.92, –9.97); ES=0.18; P<.001 | –9.58 (–11.64, –7.52); ES=0.14; P<.001 | –4.02 (–5.78, –2.26); ES=0.06; P<.001 | –5.48 (–7.08, –3.87); ES=0.08; P<.001 | –8.56 (–10.40, –6.73); ES=0.13; P<.001 |
| **3 vs 1** | –18.46 (–20.03, –16.89);  ES=0.27; P<.001 | –14.84 (–16.28, –13.40);  ES=0.22; P<.001 | –13.13 (–14.49, –11.77);  ES=0.19; P<.001 | –13.30 (–14.65, –11.94);  ES=0.19; P<.001 | –14.40 (–16.21, –12.59);  ES=0.21; P<.001 |
| **5 vs 3** | –21.31 (–23.22, –19.41);  ES=0.31; P<.001 | –16.51 (–18.44, –14.59);  ES=0.24; P<.001 | –15.04 (–17.02, –13.07);  ES=0.22; P<.001 | –12.46 (–14.60, –10.32);  ES=0.18; P<.001 | –13.20 (–16.68, –9.73); ES=0.19; P<.001 |
| **Activity Load (AU)** | | | | | |
| **1 vs –1** | –6.99 (–10.37, –3.60); ES=0.06; P<.001 | –4.63 (–7.83, –1.43); ES=0.04; P<.001 | –2.69 (–5.41, 0.02); ES=0.02; P=.0011 | –1.12 (–3.59, 1.36); ES=0.01; P=.278 | –1.86 (–4.76, 1.04); ES=0.02; P=.0614 |
| **3 vs 1** | –10.57 (–12.62, –8.51); ES=0.10; P<.001 | –10.80 (–12.98, –8.61); ES=0.10; P<.001 | –10.65 (–12.71, –8.58); ES=0.10; P<.001 | –9.33 (–11.39, –7.27); ES=0.09; P<.001 | –11.81 (–14.63, –8.98); ES=0.11; P<.001 |
| **5 vs 3** | –11.13 (–13.49, –8.78); ES=0.10; P<.001 | –9.57 (–12.43, –6.71); ES=0.09; P<.001 | –11.37 (–14.29, –8.45); ES=0.11; P<.001 | –10.21 (–13.35, –7.07); ES=0.09; P<.001 | –9.60 (–14.89, –4.30); ES=0.09; P<.001 |
| Estimates reflect dose-response contrasts between drink number and physiological or behavioral responses, with corresponding 99.9% confidence intervals, stratified by age group. ES = standardized effect size. These results correspond to the modeled associations shown in **Fig 4A-D**. | | | | | |
