# Supplementary material for: Real-world effects of alcohol on heart rate, sleep, and physical activity by age and sex
Source: PLOS Digit Health. 2026 Mar 9;5(3):e0001284. doi: 10.1371/journal.pdig.0001284 (PMC12970902; doi:10.1371/journal.pdig.0001284)
Supplement: S8 Table — (DOCX) [file pdig.0001284.s008.docx]

| **Supplemental Table 8.** Estimated age group differences in physiological and behavioral outcomes by number of drinks (within-person centered) | | | | |
| --- | --- | --- | --- | --- |
| **Number of Drinks (within-person centered)** | **20–29 vs 30–39 yrs** | **30–39 vs 40–49 yrs** | **40–49 vs 50–59 yrs** | **50–59 vs 60+ yrs** |
| **Resting Heart Rate (bpm)** | | | | |
| –1 | 0.08 (–0.13, 0.29); ES=0.02; P=.542 | –0.19 (–0.36, –0.02); ES=0.04; P<.001 | –0.08 (–0.22, 0.07); ES=0.02; P=.205 | –0.10 (–0.25, 0.04); ES=0.02; P=.038 |
| 1 | –0.03 (–0.13, 0.06); ES=0.01; P=.688 | –0.06 (–0.14, 0.02); ES=0.01; P=.033 | 0.07 (–0.01, 0.14); ES=0.01; P=.0029 | 0.08 (0.01, 0.15); ES=0.02; P=.001 |
| 3 | 0.11 (–0.03, 0.25); ES=0.02; P=.024 | 0.12 (–0.01, 0.25); ES=0.03; P=.004 | 0.33 (0.20, 0.45); ES=0.07; P<.001 | 0.63 (0.48, 0.78); ES=0.14; P<.001 |
| 5 | 0.25 (0.03, 0.46); ES=0.05; P<.001 | 0.42 (0.20, 0.63); ES=0.09; P<.001 | 0.88 (0.65, 1.11); ES=0.19; P<.001 | 0.65 (0.33, 0.96); ES=0.14; P<.001 |
| **Heart Rate Variability (ms)** | | | | |
| –1 | 1.25 (0.69, 1.81); ES=0.10; P<.001 | 1.62 (1.15, 2.08); ES=0.13; P<.001 | 0.88 (0.49, 1.28); ES=0.07; P<.001 | –0.21 (–0.59, 0.17); ES=0.02; P=.192 |
| 1 | 0.54 (0.28, 0.80); ES=0.04; P<.001 | 0.23 (0.01, 0.45); ES=0.02; P=.001 | –0.18 (–0.37, 0.02); ES=0.01; P=.004 | –0.19 (–0.39, –0.00); ES=0.02; P=.001 |
| 3 | –1.15 (–1.53, –0.77); ES=0.09; P<.001 | –1.65 (–2.00, –1.29); ES=0.13; P<.001 | –1.44 (–1.78, –1.10); ES=0.12; P<.001 | –0.60 (–0.99, –0.22); ES=0.05; P<.001 |
| 5 | –3.04 (–3.61, –2.47); ES=0.24; P<.001 | –3.49 (–4.08, –2.89); ES=0.28; P<.001 | –2.73 (–3.35, –2.11); ES=0.22; P<.001 | –0.68 (–1.47, 0.12); ES=0.05; P=.008 |
| **Sleep Duration (min)** | | | | |
| **–1** | 4.38 (1.37, 7.39); ES=0.06; P<.001 | 5.01 (2.54, 7.48); ES=0.07; P<.001 | –0.21 (–2.32, 1.91); ES<0.01; P=.996 | –3.41 (–5.53, –1.29); ES=0.05; P<.001 |
| **1** | 1.52 (0.13, 2.91); ES=0.02; P=.0002 | –0.55 (–1.73, 0.63); ES=0.01; P=.362 | 1.25 (0.21, 2.29); ES=0.02; P<.001 | –0.32 (–1.38, 0.74); ES<0.01; P=.765 |
| **3** | –2.10 (–4.12, –0.08); ES=0.03; P=.001 | –2.27 (–4.15, –0.38); ES=0.03; P<.001 | 1.42 (–0.41, 3.26); ES=0.02; P=.022 | 2.20 (–0.00, 4.40); ES=0.03; P=.001 |
| **5** | –6.90 (–9.93, –3.87); ES=0.10; P<.001 | –3.74 (–6.86, –0.61); ES=0.05; P<.001 | –1.16 (–4.48, 2.15); ES=0.02; P=.648 | 1.52 (–3.10, 6.15); ES=0.02; P=.701 |
| **Activity Load (AU)** | | | | |
| **–1** | 0.31 (–4.21, 4.82); ES<0.01; P=.999 | 1.09 (–2.77, 4.96); ES=0.01; P=.807 | 0.58 (–2.72, 3.87); ES=0.01; P=.960 | 0.31 (–3.02, 3.64); ES<0.01; P=.997 |
| **1** | –2.05 (–4.17, 0.06); ES=0.02; P=.002 | –0.84 (–2.69, 1.00); ES=0.01; P=.394 | –1.00 (–2.63, 0.63); ES=0.01; P=.125 | 1.05 (–0.63, 2.72); ES=0.01; P=.110 |
| **3** | –1.82 (–4.83, 1.18); ES=0.02; P=.129 | –0.99 (–3.91, 1.92); ES=0.01; P=.677 | –2.31 (–5.15, 0.52); ES=0.02; P=.013 | 3.53 (0.09, 6.97); ES=0.03; P=.001 |
| **5** | –3.39 (–7.85, 1.08); ES=0.03; P=.027 | 0.81 (–3.99, 5.60); ES=0.01; P=.966 | –3.47 (–8.53, 1.59); ES=0.03; P=.060 | 2.91 (–4.23, 10.06); ES=0.03; P=.507 |
| Estimates reflect age group contrasts at different drink quantities derived from estimate marginal means using generalized additive models, with corresponding 99.9% confidence intervals. ES = standardized effect size. These results correspond to the modeled associations shown in **Fig 4A-D**. | | | | |
