# Supplementary material for: Real-world effects of alcohol on heart rate, sleep, and physical activity by age and sex
Source: PLOS Digit Health. 2026 Mar 9;5(3):e0001284. doi: 10.1371/journal.pdig.0001284 (PMC12970902; doi:10.1371/journal.pdig.0001284)
Supplement: S9 Table — (DOCX) [file pdig.0001284.s009.docx]

| **Supplemental Table 9.** Estimated age group differences in physiological and behavioral outcomes by number of drinks | | | | |
| --- | --- | --- | --- | --- |
| **Number of Drinks** | **20–29 vs 30–39 yrs** | **30–39 vs 40–49 yrs** | **40–49 vs 50–59 yrs** | **50–59 vs 60+ yrs** |
| **Resting Heart Rate (bpm)** | | | | |
| 1 | 0.06 (–0.06, 0.18); ES=0.01; P=.357 | –0.13 (–0.24, –0.03); ES=0.03; P<.001 | 0.00 (–0.09, 0.09); ES=0.00; P=1.000 | 0.00 (–0.09, 0.09); ES=0.00; P=1.000 |
| 3 | –0.03 (–0.15, 0.09); ES=0.01; P=.875 | –0.02 (–0.12, 0.08); ES=0.00; P=.907 | 0.09 (0.00, 0.18); ES=0.02; P=.001 | 0.06 (–0.03, 0.15); ES=0.01; P=.042 |
| 5 | 0.15 (–0.03, 0.33); ES=0.03; P=.015 | 0.30 (0.14, 0.46); ES=0.07; P<.001 | 0.20 (0.05, 0.35); ES=0.04; P<.001 | 0.42 (0.26, 0.59); ES=0.09; P<.001 |
| 7 | 0.19 (–0.08, 0.47); ES=0.04; P=.057 | 0.35 (0.09, 0.62); ES=0.08; P<.001 | 0.92 (0.67, 1.18); ES=0.20; P<.001 | 0.36 (0.04, 0.68); ES=0.08; P<.001 |
| **Heart Rate Variability (ms)** | | | | |
| 1 | 0.84 (0.51, 1.17); ES=0.07; P<.001 | 0.89 (0.61, 1.17); ES=0.07; P<.001 | 0.29 (0.04, 0.54); ES=0.02; P<.001 | –0.04 (–0.28, 0.21); ES=0.00; P=.979 |
| 3 | –0.18 (–0.51, 0.15); ES=0.01; P=.201 | –0.28 (–0.56, –0.01); ES=0.02; P<.001 | –0.33 (–0.57, –0.09); ES=0.03; P<.001 | –0.31 (–0.54, –0.07); ES=0.02; P<.001 |
| 5 | –1.89 (–2.38, –1.40); ES=0.15; P<.001 | –2.09 (–2.53, –1.66); ES=0.17; P<.001 | –1.05 (–1.45, –0.65); ES=0.08; P<.001 | –0.57 (–1.00, –0.14); ES=0.05; P<.001 |
| 7 | –3.41 (–4.15, –2.67); ES=0.27; P<.001 | –3.17 (–3.88, –2.47); ES=0.25; P<.001 | –2.35 (–3.03, –1.68); ES=0.19; P<.001 | –0.09 (–0.91, 0.73); ES=0.01; P=.993 |
| **Sleep Duration (hrs)** | | | | |
| 1 | 0.04 (0.01, 0.07); ES=0.04; P<.001 | 0.02 (–0.01, 0.04); ES=0.01; P=.128 | 0.02 (0.00, 0.05); ES=0.02; P<.001 | –0.02 (–0.04, 0.00); ES=0.02; P=.001 |
| 3 | 0.01 (–0.02, 0.04); ES=0.01; P=.923 | –0.01 (–0.03, 0.02); ES=0.01; P=.863 | 0.01 (–0.01, 0.03); ES=0.01; P=.773 | –0.02 (–0.04, 0.01); ES=0.01; P=.044 |
| 5 | –0.07 (–0.12, –0.03); ES=0.07; P<.001 | –0.03 (–0.06, 0.01); ES=0.02; P=.042 | 0.02 (–0.01, 0.06); ES=0.02; P=.040 | 0.03 (–0.01, 0.07); ES=0.02; P=.037 |
| 7 | –0.15 (–0.21, –0.09); ES=0.13; P<.001 | –0.08 (–0.14, –0.03); ES=0.07; P<.001 | –0.00 (–0.06, 0.05); ES<0.01; P=1.000 | 0.01 (–0.06, 0.09); ES=0.01; P=.961 |
| **Activity Load (AU)** | | | | |
| 1 | –2.04 (–4.74, 0.67); ES=0.02; P=.029 | –0.35 (–2.70, 2.00); ES<0.01; P=.979 | –0.82 (–2.91, 1.26); ES=0.01; P=.543 | 1.97 (–0.08, 4.03); ES=0.02; P=.002 |
| 3 | –1.92 (–4.41, 0.56); ES=0.02; P=.023 | –0.78 (–2.96, 1.39); ES=0.01; P=.630 | –0.84 (–2.77, 1.08); ES=0.01; P=.438 | 0.09 (–1.88, 2.06); ES<0.01; P=1.000 |
| 5 | –2.01 (–5.42, 1.40); ES=0.02; P=.151 | –1.33 (–4.49, 1.84); ES=0.01; P=.485 | –1.41 (–4.38, 1.55); ES=0.01; P=.349 | 0.75 (–2.62, 4.12); ES=0.01; P=.910 |
| 7 | –3.32 (–8.07, 1.44); ES=0.03; P=.052 | –0.89 (–5.61, 3.83); ES=0.01; P=.949 | –3.54 (–8.18, 1.10); ES=0.03; P=.026 | 3.39 (–2.66, 9.43); ES=0.03; P=.188 |
| Estimates reflect age group contrasts at different drink quantities derived from estimate marginal means using generalized additive models, with corresponding 99.9% confidence intervals. ES = standardized effect size. These results correspond to the modeled associations shown in **S4** **Fig**. | | | | |
