# Supplementary material for: Real-world effects of alcohol on heart rate, sleep, and physical activity by age and sex
Source: PLOS Digit Health. 2026 Mar 9;5(3):e0001284. doi: 10.1371/journal.pdig.0001284 (PMC12970902; doi:10.1371/journal.pdig.0001284)
Supplement: S10 Table — (DOCX) [file pdig.0001284.s010.docx]

| **Supplemental Table 10.** Estimated differences in physiological and behavioral outcomes by time between last alcoholic drink and bedtime (within-person centered) by age group | | | | | |
| --- | --- | --- | --- | --- | --- |
| **Time from Last Drink to Bed (within-person centered)** | **20–29 yrs** | **30–39 yrs** | **40–49 yrs** | **50–59 yrs** | **60+ yrs** |
| **Resting Heart Rate (bpm)** | | | | | |
| -60 vs -180 min | -0.65 (-0.87, -0.43); ES=0.14; P<.001 | -0.71 (-0.91, -0.52); ES=0.16; P<.001 | -0.62 (-0.80, -0.44); ES=0.14; P<.001 | -0.45 (-0.61, -0.28); ES=0.10; P<.001 | -0.45 (-0.61, -0.29); ES=0.10; P<.001 |
| 60 vs -60 min | -1.19 (-1.33, -1.06); ES=0.26; P<.001 | -1.01 (-1.12, -0.90); ES=0.22; P<.001 | -0.76 (-0.86, -0.67); ES=0.17; P<.001 | -0.65 (-0.74, -0.56); ES=0.14; P<.001 | -0.50 (-0.58, -0.41); ES=0.11; P<.001 |
| 180 vs 60 min | -0.66 (-0.85, -0.48); ES=0.15; P<.001 | -0.54 (-0.70, -0.39); ES=0.12; P<.001 | -0.56 (-0.70, -0.42); ES=0.12; P<.001 | -0.43 (-0.56, -0.30); ES=0.09; P<.001 | -0.43 (-0.56, -0.31); ES=0.10; P<.001 |
| 300 vs 180 min | -0.36 (-0.61, -0.10); ES=0.08; P<.001 | -0.40 (-0.62, -0.17); ES=0.09; P<.001 | -0.36 (-0.56, -0.15); ES=0.08; P<.001 | -0.51 (-0.70, -0.32); ES=0.11; P<.001 | -0.38 (-0.56, -0.21); ES=0.08; P<.001 |
| 420 vs 300 min | 0.10 (-0.28, 0.49); ES=0.02; P=.824 | -0.13 (-0.48, 0.21); ES=0.03; P=.528 | -0.04 (-0.35, 0.28); ES=0.01; P=.995 | -0.26 (-0.56, 0.03); ES=0.06; P=.005 | -0.21 (-0.48, 0.07); ES=0.05; P=.022 |
| **Heart Rate Variability (ms)** | | | | | |
| **-60 vs -180 min** | 2.33 (1.73, 2.93); ES=0.19; P<.001 | 1.70 (1.19, 2.22); ES=0.14; P<.001 | 0.68 (0.24, 1.13); ES=0.05; P<.001 | 0.48 (0.16, 0.80); ES=0.04; P<.001 | 0.54 (0.43, 0.66); ES=0.04; P<.001 |
| **60 vs -60 min** | 3.74 (3.38, 4.10); ES=0.30; P<.001 | 2.15 (1.85, 2.44); ES=0.17; P<.001 | 1.15 (0.90, 1.39); ES=0.09; P<.001 | 0.63 (0.45, 0.81); ES=0.05; P<.001 | 0.54 (0.43, 0.66); ES=0.04; P<.001 |
| **180 vs 60 min** | 1.69 (1.18, 2.19); ES=0.14; P<.001 | 1.18 (0.76, 1.60); ES=0.09; P<.001 | 0.95 (0.61, 1.29); ES=0.08; P<.001 | 0.64 (0.41, 0.87); ES=0.05; P<.001 | 0.54 (0.43, 0.66); ES=0.04; P<.001 |
| **300 vs 180 min** | 1.29 (0.57, 2.00); ES=0.10; P<.001 | 1.15 (0.56, 1.74); ES=0.09; P<.001 | 0.92 (0.44, 1.40); ES=0.07; P<.001 | 0.75 (0.44, 1.05); ES=0.06; P<.001 | 0.54 (0.43, 0.66); ES=0.04; P<.001 |
| **420 vs 300 min** | 0.66 (-0.43, 1.74); ES=0.05; P=.112 | 1.44 (0.54, 2.34); ES=0.12; P<.001 | 1.05 (0.30, 1.79); ES=0.08; P<.001 | 0.86 (0.37, 1.35); ES=0.07; P<.001 | 0.54 (0.42, 0.66); ES=0.04; P<.001 |
| **Sleep Duration (min)** | | | | | |
| -60 vs -180 min | -7.87 (−11.39, −4.34); ES=0.12; P<.001 | -9.20 (−12.40, −6.01); ES=0.14; P<.001 | -9.74 (−12.76, −6.71); ES=0.14; P<.001 | -14.13 (−16.90, −11.35); ES=0.21; P<.001 | -15.62 (−18.79, −12.46); ES=0.23; P<.001 |
| 60 vs -60 min | -9.21 (−11.30, −7.13); ES=0.14; P<.001 | -13.97 (−15.77, −12.18); ES=.21; P<.001 | -19.56 (−21.13, −17.98); ES=0.29; P<.001 | -21.36 (−22.77, −19.95); ES=0.31; P<.001 | -26.03 (−27.57, −24.49); ES=0.38; P<.001 |
| 180 vs 60 min | -4.35 (−7.37, −1.33); ES=0.06; P<.001 | -7.25 (−9.94, −4.56); ES=0.11; P<.001 | -7.53 (−9.98, −5.07); ES=0.11; P<.001 | -11.30 (−13.56, −9.05); ES=0.17; P<.001 | -17.33 (−19.91, −14.75); ES=0.25; P<.001 |
| 300 vs 180 min | 1.26 (−3.22, 5.74); ES=0.02; P=.792 | -2.07 (−6.14, 2.00); ES=0.03; P=.242 | -4.36 (−8.21, −0.51); ES=0.06; P<.001 | -7.02 (−10.55, −3.49); ES=0.10; P<.001 | -7.88 (−12.03, −3.73); ES=0.12; P<.001 |
| 420 vs 300 min | -27.49 (−34.76, −20.23); ES=0.40; P<.001 | -22.24 (−28.86, −15.61); ES=0.33; P<.001 | -27.75 (−34.04, −21.46); ES=0.41; P<.001 | -31.61 (−37.53, −25.69); ES=0.46; P<.001 | -40.11 (−47.22, −33.00); ES=0.59; P<.001 |
| **Activity Load (AU)** | | | | | |
| **-60 vs -180 min** | 2.33 (−1.96, 6.61); ES=0.02; P=.192 | 1.34 (−1.77, 4.46); ES=0.01; P=.418 | 2.52 (−1.30, 6.35); ES=0.02; P=.067 | 3.33 (0.02, 6.63); ES=0.03; P=.001 | 2.61 (−1.62, 6.84); ES=0.02; P=.101 |
| **60 vs -60 min** | −0.22 (−2.73, 2.30); ES=0.00; P=.999 | 0.31 (−1.49, 2.11); ES=0.00; P=.971 | −0.80 (−2.90, 1.29); ES=0.00; P=.545 | −0.37 (−2.17, 1.42); ES=0.00; P=.938 | −1.13 (−3.36, 1.11); ES=0.00; P=.257 |
| **180 vs 60 min** | 0.41 (−2.78, 3.61); ES=0.00; P=.992 | −1.05 (−3.19, 1.09); ES=0.01; P=.281 | −2.23 (−5.09, 0.63); ES=0.02; P=.018 | −2.00 (−4.45, 0.44); ES=0.02; P=.011 | −3.20 (−6.58, 0.18); ES=0.03; P=.002 |
| **300 vs 180 min** | 0.29 (−3.91, 4.49); ES=0.00; P=1.000 | −2.10 (−4.94, 0.73); ES=0.02; P=.028 | 0.22 (−3.77, 4.22); ES=0.00; P=1.000 | −1.99 (−5.35, 1.36); ES=0.02; P=.125 | 0.16 (−4.79, 5.10); ES=0.00; P=1.000 |
| **420 vs 300 min** | −3.75 (−10.37, 2.86); ES=0.03; P=.158 | −2.95 (−7.46, 1.56); ES=0.03; P=.072 | −2.85 (−9.08, 3.39); ES=0.03; P=.359 | −2.82 (−8.17, 2.53); ES=0.03; P=.219 | −3.95 (−11.72, 3.82); ES=0.04; P=.249 |
| Estimates reflect dose-response contrasts between drink timing and physiological or behavioral responses, with corresponding 99.9% confidence intervals, stratified by age group. ES = standardized effect size. These results correspond to the modeled associations shown in **Fig 5A-D**. | | | | | |
