# Supplementary material for: Real-world effects of alcohol on heart rate, sleep, and physical activity by age and sex
Source: PLOS Digit Health. 2026 Mar 9;5(3):e0001284. doi: 10.1371/journal.pdig.0001284 (PMC12970902; doi:10.1371/journal.pdig.0001284)
Supplement: S11 Table — (DOCX) [file pdig.0001284.s011.docx]

| **Supplemental Table 11.** Estimated age group differences in physiological and behavioral outcomes at varying times from last alcoholic drink to bedtime (within-person centered) | | | | |
| --- | --- | --- | --- | --- |
| **Time from Last Drink to Bed (within-person centered)** | **20–29 vs 30–39 yrs** | **30–39 vs 40–49 yrs** | **40–49 vs 50–59 yrs** | **50–59 vs 60+ yrs** |
| **Resting Heart Rate (bpm)** | | | | |
| −180 min | 0.13 (−0.14, 0.40); ES=0.03; P=.336 | 0.20 (−0.05, 0.45); ES=0.04; P=.017 | 0.33 (0.09, 0.56); ES=0.07; P < .001 | 0.12 (−0.11, 0.34); ES=0.03; P=.255 |
| −60 min | 0.20 (0.09, 0.30); ES=0.04; P <.001 | 0.11 (0.02, 0.19); ES=0.02; P <.001 | 0.15 (0.07, 0.23); ES=0.03; P <.001 | 0.12 (0.04, 0.19); ES=0.03; P <.001 |
| 60 min | 0.01 (−0.12, 0.14); ES<.01; P =.999 | −0.14 (−0.25, −0.02); ES=0.03; P=.001 | 0.04 (−0.06, 0.14); ES=0.01; P=.495 | −0.04 (−0.13, 0.06); ES=0.01; P=.575 |
| 180 min | −0.11 (−0.34, 0.12); ES=0.02; P=.322 | −0.13 (−0.33, 0.08); ES=0.03; P=.113 | −0.09 (−0.27, 0.10); ES=0.02; P=.378 | −0.03 (−0.21, 0.15); ES=0.01; P=.963 |
| 300 min | −0.07 (−0.40, 0.25); ES=0.02; P=.908 | −0.16 (−0.46, 0.14); ES=0.04; P=.035 | 0.07 (−0.22, 0.35); ES=0.01; P=.896 | −0.16 (−0.44, 0.12); ES=0.03; P=.177 |
| 420 min | 0.16 (−0.34, 0.67); ES=0.04; P=.713 | −0.26 (−0.73, 0.21); ES=0.06; P=.196 | 0.29 (−0.15, 0.74); ES=0.06; P=.077 | −0.21 (−0.66, 0.23); ES=0.05; P=.342 |
| **Heart Rate Variability (ms)** | | | | |
| −180 min | −1.90 (−2.63, −1.17); ES=0.15; P<.001 | −1.59 (−2.24, −0.95); ES=0.13; P<.001 | −0.63 (−1.18, −0.08); ES=0.05; P=.001 | −0.07 (−0.47, 0.34); ES<.01; P=.970 |
| −60 min | −1.27 (−1.56, −0.99); ES=0.10; P<.001 | −0.58 (−0.81, −0.34); ES=0.05; P<.001 | −0.43 (−0.63, −0.23); ES=0.03; P<.001 | −0.13 (−0.30, 0.05); ES=0.01; P=.046 |
| 60 min | 0.32 (−0.05, 0.69); ES=0.03; P=.007 | 0.42 (0.12, 0.73); ES=0.03; P<.001 | 0.08 (−0.16, 0.33); ES=0.01; P=.650 | −0.04 (−0.23, 0.16); ES<.01; P=.950 |
| 180 min | 0.83 (0.21, 1.45); ES=0.07; P<.001 | 0.65 (0.12, 1.18); ES=0.05; P<.001 | 0.40 (−0.04, 0.83); ES=0.03; P=.005 | 0.06 (−0.28, 0.41); ES<.01; P=.960 |
| 300 min | 0.96 (0.07, 1.86); ES=0.08; P<.001 | 0.88 (0.10, 1.66); ES=0.07; P<.001 | 0.57 (−0.09, 1.23); ES=0.05; P=.007 | 0.27 (−0.26, 0.80); ES=0.02; P=.290 |
| 420 min | 0.17 (−1.19, 1.54); ES=0.01; P=.99 | 1.27 (0.06, 2.49); ES=0.10; P=.001 | 0.76 (−0.31, 1.83); ES=0.06; P=.045 | 0.59 (−0.24, 1.41); ES=0.05; P=.048 |
| **Sleep Duration (min)** | | | | |
| −180 min | −4.00 (−6.63, −1.34); ES=0.06; P=.003 | −3.33 (−6.27, 0.68); ES=0.05; P=.011 | −4.27 (−8.04, −0.49); ES=0.06; P<.001 | −4.70 (−8.61, −0.79); ES=0.07; P<.001 |
| −60 min | −2.66 (−4.31, −1.01); ES=0.04; P<.001 | −2.79 (−4.23, −1.36); ES=0.04; P<.001 | 0.12 (−1.16, 1.41); ES=0.01; P=.995 | −3.20 (−4.50, −1.90); ES=0.05; P<.001 |
| 60 min | 2.10 (0.00, 4.21); ES=0.03; P=.001 | 2.79 (1.00, 4.59); ES=0.04; P<.001 | 1.93 (0.35, 3.50); ES=0.03; P<.001 | 1.47 (−0.07, 3.01); ES=0.02; P=.002 |
| 180 min | 5.01 (1.35, 8.66); ES=0.07; P<.001 | 3.07 (−0.26, 6.39); ES=0.04; P=.003 | 5.70 (2.63, 8.78); ES=0.08; P<.001 | 7.50 (4.28, 10.71); ES=0.11; P<.001 |
| 300 min | 8.33 (2.95, 13.71); ES=0.12; P<.001 | 5.36 (0.32, 10.40); ES=0.08; P<.001 | 8.37 (3.58, 13.15); ES=0.12; P<.001 | 8.35 (3.31, 13.39); ES=0.12; P<.001 |
| 420 min | 3.08 (−5.77, 11.92); ES=0.05; P=.659 | 10.87 (2.56, 19.19); ES=0.16; P<.001 | 12.22 (4.23, 20.22); ES=0.18; P<.001 | 16.85 (8.21, 25.49); ES=0.25; P<.001 |
| **Activity Load (AU)** | | | | |
| **−180 min** | −3.23 (−8.54, 2.07); ES=0.03; P=.125 | 0.24 (−4.74, 5.21); ES=0.00; P=.999 | −0.09 (−5.06, 4.87); ES=0.00; P=.999 | 0.33 (−4.92, 5.58); ES<0.01; P=.999 |
| **−60 min** | −2.25 (−4.40, −0.10); ES=0.02; P=.001 | −0.94 (−2.83, 0.94); ES=0.01; P=.293 | −0.90 (−2.67, 0.88); ES=0.01; P=.282 | 1.05 (−0.79, 2.89); ES=0.01; P=.173 |
| **60 min** | −2.77 (−5.47, −0.07); ES=0.03; P=.001 | 0.17 (−2.16, 2.49); ES=0.00; P=.999 | −1.33 (−3.51, 0.85); ES=0.01; P=.126 | 1.80 (−0.40, 4.01); ES=0.02; P=.013 |
| **180 min** | −1.30 (−5.60, 2.99); ES=0.01; P=.763 | 1.35 (−2.59, 5.29); ES=0.01; P=.674 | −1.56 (−5.51, 2.39); ES=0.01; P=.543 | 3.00 (−1.29, 7.29); ES=0.03; P=.052 |
| **300 min** | 1.09 (−5.03, 7.20); ES=0.01; P=.959 | −0.98 (−6.82, 4.87); ES=0.01; P=.967 | 0.66 (−5.30, 6.61); ES=0.01; P=.993 | 0.85 (−5.72, 7.42); ES=0.01; P=.987 |
| **420 min** | 0.28 (−9.75, 10.31); ES=0.00; P=.999 | −1.07 (−10.64, 8.49); ES=0.01; P=.992 | 0.63 (−8.94, 10.20); ES=0.01; P=.990 | 1.98 (−8.59, 12.55); ES=0.02; P=.950 |
| Estimates reflect age group contrasts at different drink timings derived from estimate marginal means using generalized additive models, with corresponding 99.9% confidence intervals. ES = standardized effect size. These results correspond to the modeled associations shown in **Fig 5A-D**. | | | | |
