# Supplementary material for: Real-world effects of alcohol on heart rate, sleep, and physical activity by age and sex
Source: PLOS Digit Health. 2026 Mar 9;5(3):e0001284. doi: 10.1371/journal.pdig.0001284 (PMC12970902; doi:10.1371/journal.pdig.0001284)
Supplement: S12 Table — (DOCX) [file pdig.0001284.s012.docx]

| **Supplemental Table 12.** Estimated sleep tertile differences in physiological and behavioral outcomes by number of drinks (within-person centered) | | | |
| --- | --- | --- | --- |
| **Number of Drinks (within‑person)** | **Low vs. Med** | **Low vs. High** | **Med vs. High** |
| **Resting Heart Rate (bpm)** | | | |
| **−1** | **0.80 (0.68, 0.91); ES=0.18; P<.001** | **1.17 (1.05, 1.28); ES=0.26; P<.001** | **0.37 (0.26, 0.48); ES=0.08; P<.001** |
| **1** | **0.94 (0.88, 0.99); ES=0.21; P<.001** | **1.46 (1.40, 1.52); ES=0.33; P<.001** | **0.52 (0.47, 0.58); ES=0.12; P<.001** |
| **3** | **1.56 (1.46, 1.66); ES=0.35; P<.001** | **2.39 (2.30, 2.49); ES=0.53; P<.001** | **0.83 (0.73, 0.93); ES=0.19; P<.001** |
| **5** | **1.98 (1.81, 2.15); ES=0.44; P<.001** | **2.90 (2.74, 3.07); ES=0.65; P<.001** | **0.93 (0.75, 1.11); ES=0.21; P<.001** |
| **Heart Rate Variability (ms)** | | | |
| −1 | −1.94 (−2.25, −1.63); ES=0.16; P<.001 | −3.08 (−3.40, −2.77); ES=0.25; P<.001 | −1.15 (−1.45, −0.85); ES=0.09; P<.001 |
| 1 | −2.06 (−2.21, −1.90); ES=0.17; P<.001 | −3.50 (−3.65, −3.35); ES=0.28; P<.001 | −1.44 (−1.59, −1.30); ES=0.12; P<.001 |
| 3 | −3.35 (−3.61, −3.09); ES=0.27; P<.001 | −5.32 (−5.57, −5.06); ES=0.43; P<.001 | −1.97 (−2.24, −1.70); ES=0.16; P<.001 |
| 5 | −4.63 (−5.08, −4.19); ES=0.37; P<.001 | −7.19 (−7.62, −6.76); ES=0.58; P<.001 | −2.56 (−3.04, −2.08); ES=0.21; P<.001 |
| **Activity Load (AU)** | | | |
| −1 | −4.58 (−7.26, −1.89); ES=0.04; P<.001 | −2.90 (−5.60, −0.21); ES=0.03; P=.0004 | 1.67 (−0.92, 4.27); ES=0.02; P=.055 |
| 1 | −4.75 (−6.07, −3.43); ES=0.04; P<.001 | −3.81 (−5.13, −2.49); ES=0.04; P<.001 | 0.94 (−0.34, 2.22); ES=0.01; P=.024 |
| 3 | −6.99 (−9.22, −4.76); ES=0.06; P<.001 | −5.21 (−7.39, −3.02); ES=0.05; P<.001 | 1.78 (−0.50, 4.07); ES=0.02; P=.014 |
| 5 | −7.21 (−10.98, −3.45); ES=0.07; P<.001 | −6.03 (−9.69, −2.37); ES=0.06; P<.001 | 1.19 (−2.85, 5.23); ES=0.01; P=.543 |
| Estimates reflect sleep level contrasts at different drink quantities derived from estimate marginal means using generalized additive models. Nights after drinking were stratified into low (≤ −0.36 hours), moderate (> −0.36 to ≤ 0.49 hours), and high (> 0.49 hours) tertiles based on deviations from individuals’ personal average sleep, with higher values reflecting more sleep than usual. Results represent differences in physiological and behavioral responses with 99.9% confidence intervals. ES = standardized effect size. These results correspond to the modeled associations shown in **Fig 6A-C**. | | | |
