# Supplementary material for: Real-world effects of alcohol on heart rate, sleep, and physical activity by age and sex
Source: PLOS Digit Health. 2026 Mar 9;5(3):e0001284. doi: 10.1371/journal.pdig.0001284 (PMC12970902; doi:10.1371/journal.pdig.0001284)
Supplement: S13 Table — (DOCX) [file pdig.0001284.s013.docx]

| **Supplemental Table 13.** Estimated activity tertile differences in physiological and behavioral outcomes by number of drinks (within-person centered) | | | |
| --- | --- | --- | --- |
| **Number of Drinks (within-person centered)** | **Low vs. Med** | **Low vs. High** | **Med vs. High** |
| **Resting Heart Rate (bpm)** | | | |
| −1 | −0.02 (−0.14, 0.10); ES<0.01; P<.842 | −0.48 (−0.60, −0.36); ES=0.11; P<.001 | −0.46 (−0.58, −0.34); ES=0.10; P<.001 |
| 1 | −0.10 (−0.16, −0.04); ES=0.02; P<.001 | −0.38 (−0.43, −0.32); ES=0.08; P<.001 | −0.28 (−0.34, −0.22); ES=0.06; P<.001 |
| 3 | −0.22 (−0.32, −0.11); ES=0.05; P<.001 | −0.51 (−0.61, −0.40); ES=0.11; P<.001 | −0.29 (−0.40, −0.18); ES=0.06; P<.001 |
| 5 | −0.39 (−0.57, −0.21); ES=0.09; P<.001 | −0.67 (−0.86, −0.49); ES=0.15; P<.001 | −0.28 (−0.47, −0.10); ES=0.06; P<.001 |
| **Heart Rate Variability (ms)** | | | |
| −1 | 0.68 (0.34, 1.02); ES=0.05; P<.001 | 1.14 (0.81, 1.48); ES=0.09; P<.001 | 0.46 (0.12, 0.80); ES=0.04; P<.001 |
| 1 | 0.36 (0.19, 0.52); ES=0.03; P<.001 | 1.05 (0.88, 1.21); ES=0.08; P<.001 | 0.69 (0.52, 0.86); ES=0.06; P<.001 |
| 3 | 0.23 (−0.06, 0.52); ES=0.02; P=.011 | 1.35 (1.06, 1.63); ES=0.11; P<.001 | 1.11 (0.81, 1.41); ES=0.09; P<.001 |
| 5 | −0.11 (−0.60, 0.38); ES=0.01; P=.704 | 2.05 (1.56, 2.54); ES=0.16; P<.001 | 2.16 (1.65, 2.67); ES=0.17; P<.001 |
| **Sleep Duration (min)** | | | |
| −1 | 0.98 (−0.79, 2.75); ES=0.01; P=.114 | 1.74 (−0.05, 3.53); ES=0.03; P=.001 | 0.76 (−1.03, 2.55); ES=0.01; P=.280 |
| 1 | 0.72 (−0.17, 1.60); ES=0.01; P=.009 | −0.35 (−1.23, 0.53); ES=−0.01; P=.315 | −1.07 (−1.97, −0.17); ES=−0.02; P<.001 |
| 3 | 0.79 (−0.72, 2.30); ES=0.01; P=.144 | 1.65 (0.13, 3.17); ES=0.02; P<.001 | 0.86 (−0.71, 2.44); ES=0.01; P=.119 |
| 5 | 1.66 (−0.90, 4.22); ES=0.02; P=.052 | 4.26 (1.67, 6.85); ES=0.06; P<.001 | 2.60 (−0.09, 5.29); ES=0.04; P=.002 |
| Estimates reflect exercise level contrasts at different drink quantities derived from estimate marginal means using generalized additive models. Exercise before drinking was stratified into low(≤ −41.81 AU), moderate (> −41.81 to ≤ 6.22 AU), and high (> 6.22 AU) tertiles based on deviations from individuals’ personal average exercise levels, with higher values reflecting more exercise than usual. Results represent differences in physiological and behavioral responses with 99.9% confidence intervals. ES = standardized effect size. These results correspond to the modeled associations shown in **Fig 6D-F**. | | | |
