# Supplementary material for: Real-world effects of alcohol on heart rate, sleep, and physical activity by age and sex
Source: PLOS Digit Health. 2026 Mar 9;5(3):e0001284. doi: 10.1371/journal.pdig.0001284 (PMC12970902; doi:10.1371/journal.pdig.0001284)
Supplement: S14 Table — (DOCX) [file pdig.0001284.s014.docx]

| **Supplemental Table 14.**  Estimated hydration differences in physiological and behavioral outcomes by number of drinks (within-person centered) | | | |
| --- | --- | --- | --- |
| **Number of Drinks (within-person centered)** | **False – True Estimate (99.9% CI)** | **Effect Size (ES)** | **P-Value** |
| **Resting Heart Rate (bpm)** | | | |
| –1 | –0.08 (–0.17, 0.01) | 0.02 | .005 |
| 1 | 0.08 (0.03, 0.12) | 0.02 | <.001 |
| 3 | 0.15 (0.07, 0.23) | 0.03 | <.001 |
| 5 | 0.32 (0.18, 0.47) | 0.07 | <.001 |
| **Heart Rate Variability (ms)** | | | |
| –1 | 0.31 (0.06, 0.57) | 0.02 | P<.001 |
| 1 | 0.02 (–0.10, 0.14) | 0.00 | P=.611 |
| 3 | –0.22 (–0.44, 0.00) | 0.02 | P=.001 |
| 5 | –0.89 (–1.28, –0.51) | 0.07 | P<.001 |
| **Sleep Duration (min)** | | | |
| –1 | –0.13 (–1.48, 1.21) | 0.00 | P=.744 |
| 1 | –0.75 (–1.41, –0.09) | 0.01 | P<.001 |
| 3 | –1.66 (–2.82, –0.50) | 0.02 | P<.001 |
| 5 | –2.70 (–4.70, –0.70) | 0.04 | P<.001 |
| **Activity Load (AU)** | | | |
| –1 | –1.43 (–3.56, 0.70) | 0.01 | P=.027 |
| 1 | –1.34 (–2.38, –0.31) | 0.01 | P<.001 |
| 3 | –1.56 (–3.37, 0.25) | 0.01 | P=.005 |
| 5 | –1.00 (–4.11, 2.10) | 0.01 | P=.289 |
| Estimates reflect False – True hydration status contrasts derived from estimate marginal means using generalized additive models, with corresponding 99.9% confidence intervals. True represents a night where an individual reported hydration. ES = standardized effect size. These results correspond to the modeled associations shown in **S6** **Fig**. | | | |
