# Supplementary material for: Real-world effects of alcohol on heart rate, sleep, and physical activity by age and sex
Source: PLOS Digit Health. 2026 Mar 9;5(3):e0001284. doi: 10.1371/journal.pdig.0001284 (PMC12970902; doi:10.1371/journal.pdig.0001284)
Supplement: S1 Fig — (DOCX) [file pdig.0001284.s015.docx]

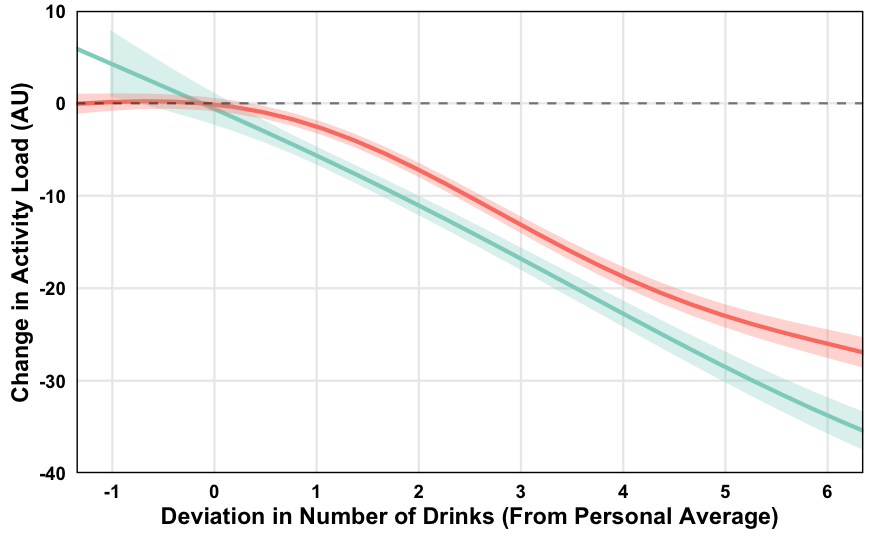

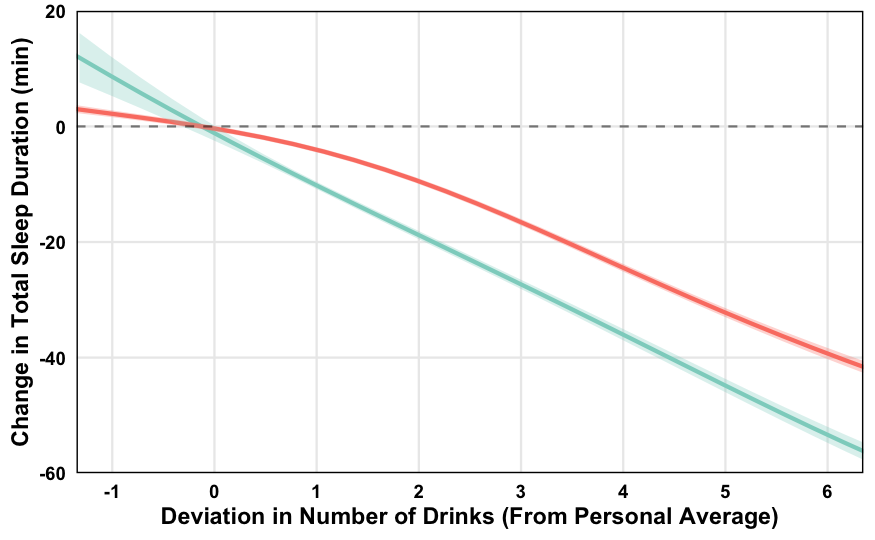

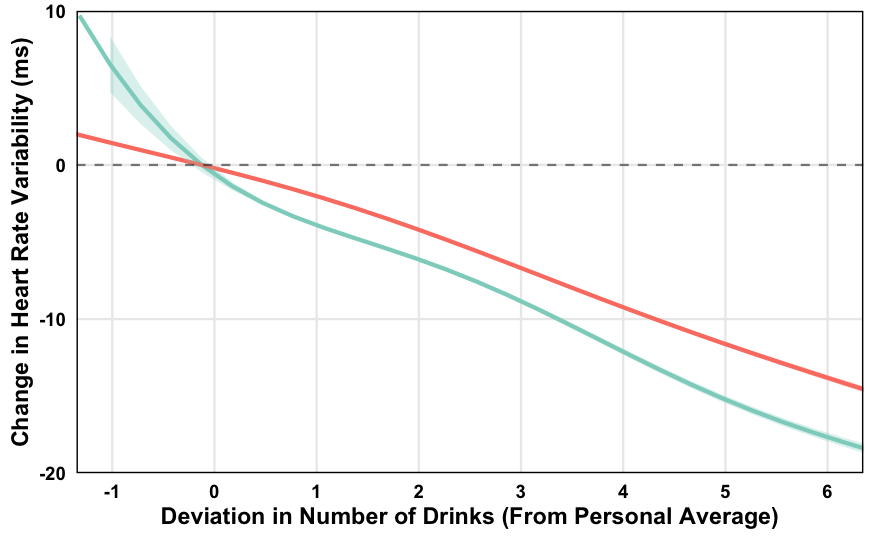

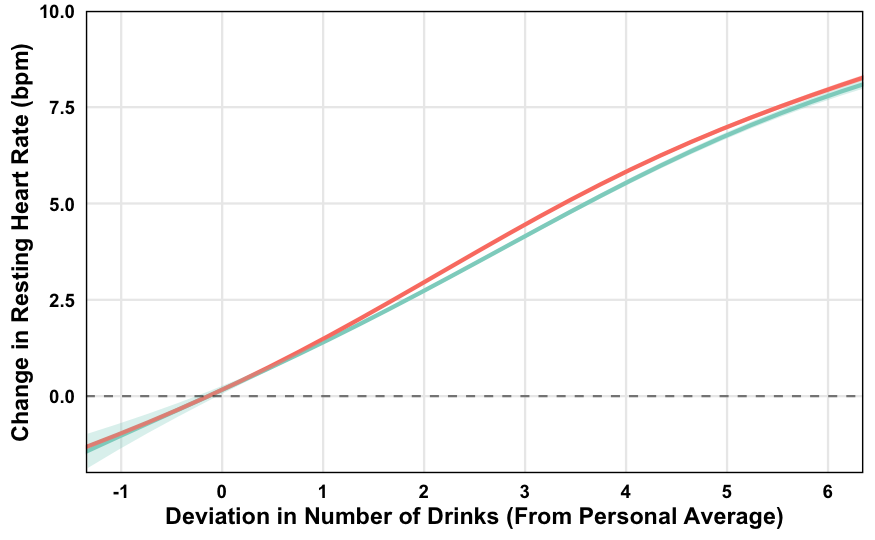

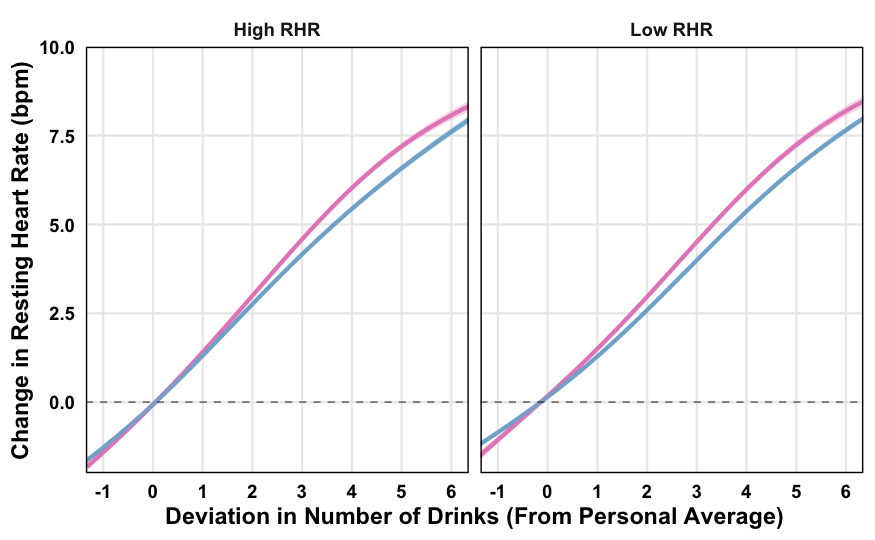

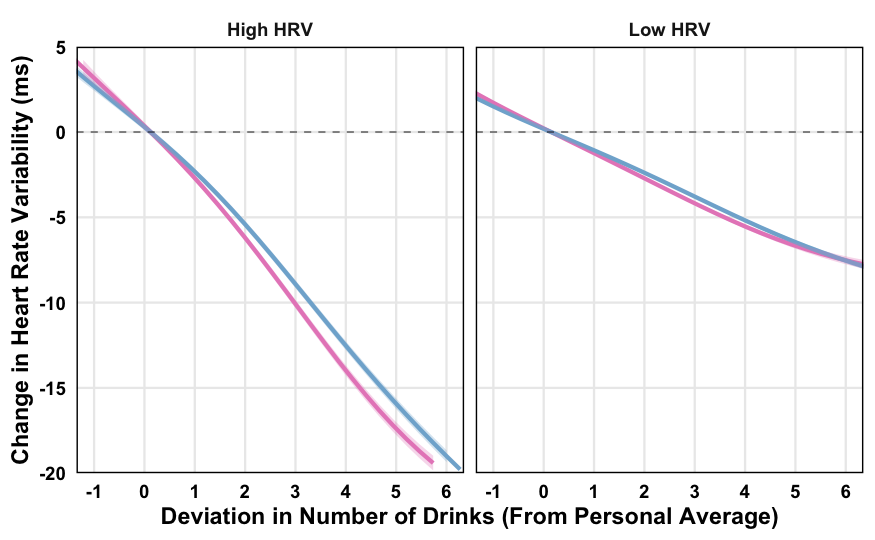

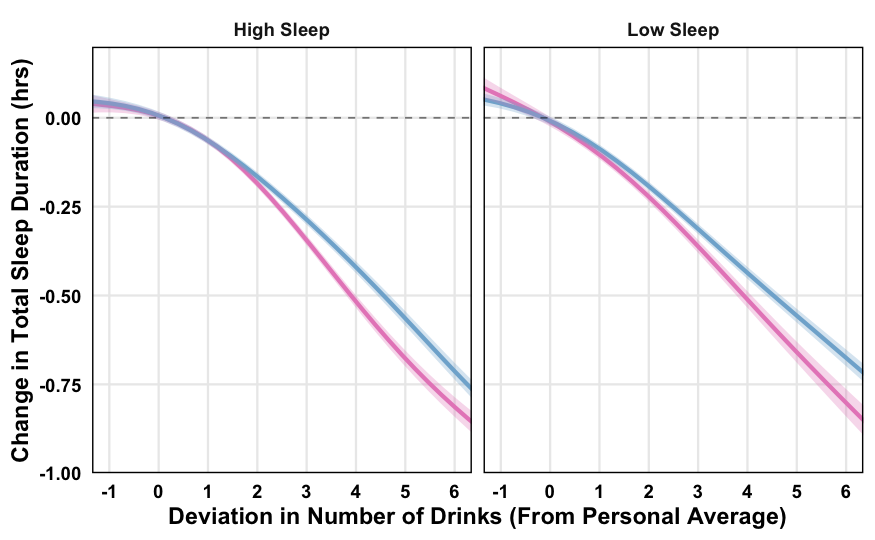

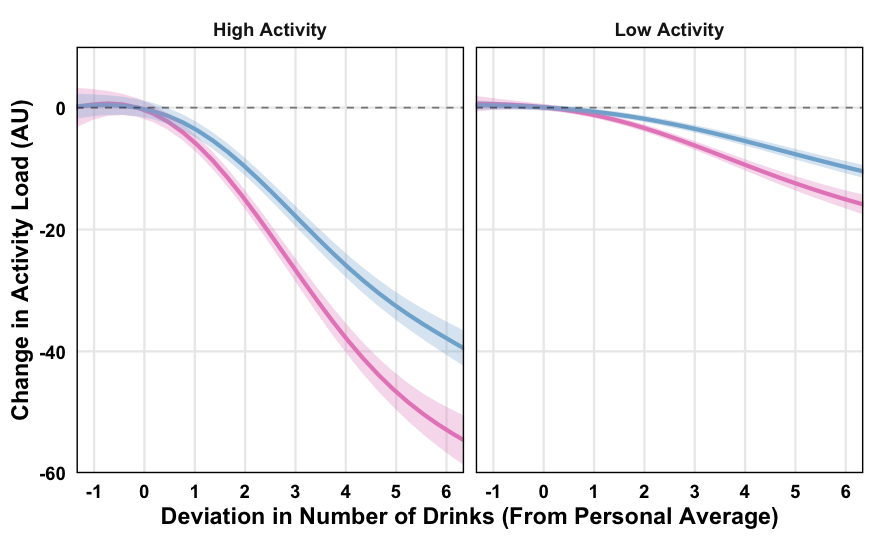


**Drinking Frequency**


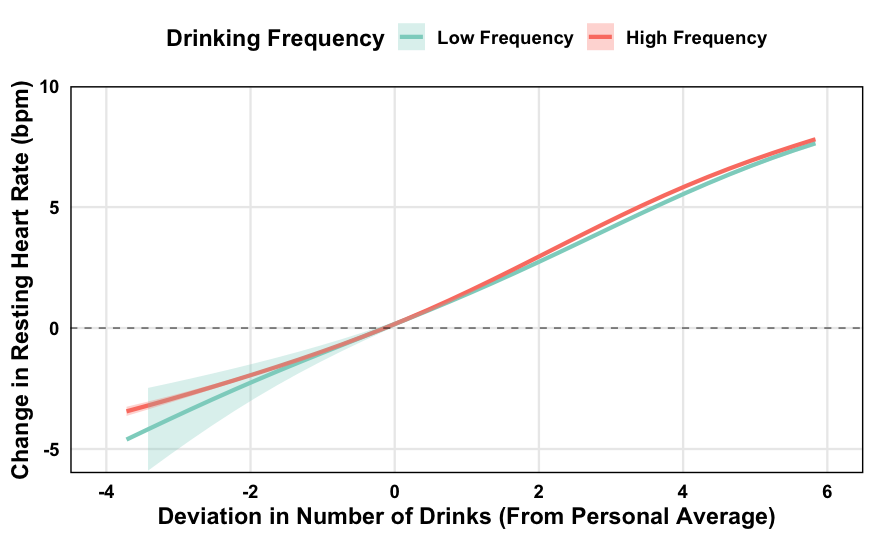


**A)**

**B)**

**C)**

**D)**

**Figure S1.** Moderation of within-person associations between deviation in alcoholic drink number and physiological and behavioral outcomes by drinking frequency. Participants were stratified into “Low” and ”High” frequency drinking groups based on a median split (23.75%) of the proportion of nights they reported consuming alcohol. Generalized additive models estimated changes in resting heart rate (**A**), heart rate variability (**B**), sleep duration (**C**), and next-day activity (**D**) based on deviations from individuals’ personal average number of drinks. Contrasts between drinking frequency groups at different drink quantities are presented in **S1 Table**.
