# Supplementary material for: Real-world effects of alcohol on heart rate, sleep, and physical activity by age and sex
Source: PLOS Digit Health. 2026 Mar 9;5(3):e0001284. doi: 10.1371/journal.pdig.0001284 (PMC12970902; doi:10.1371/journal.pdig.0001284)
Supplement: S2 Fig — (DOCX) [file pdig.0001284.s016.docx]

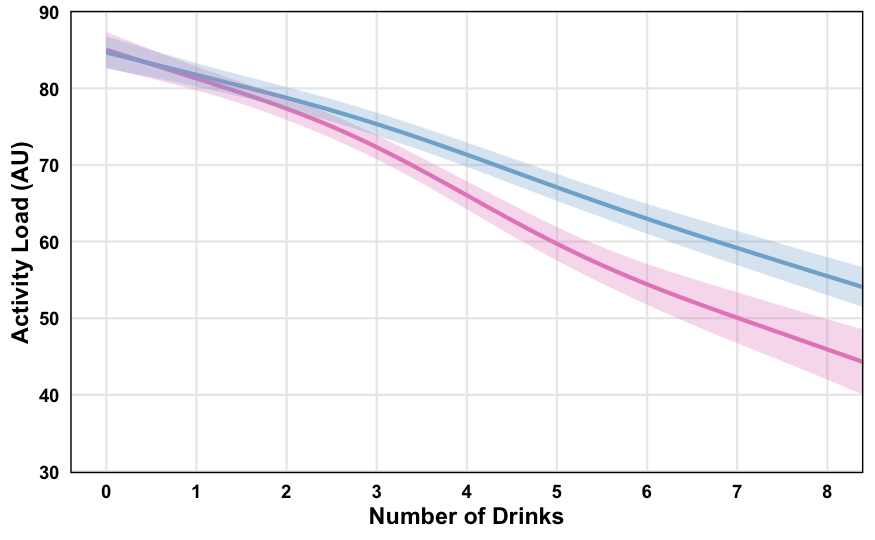

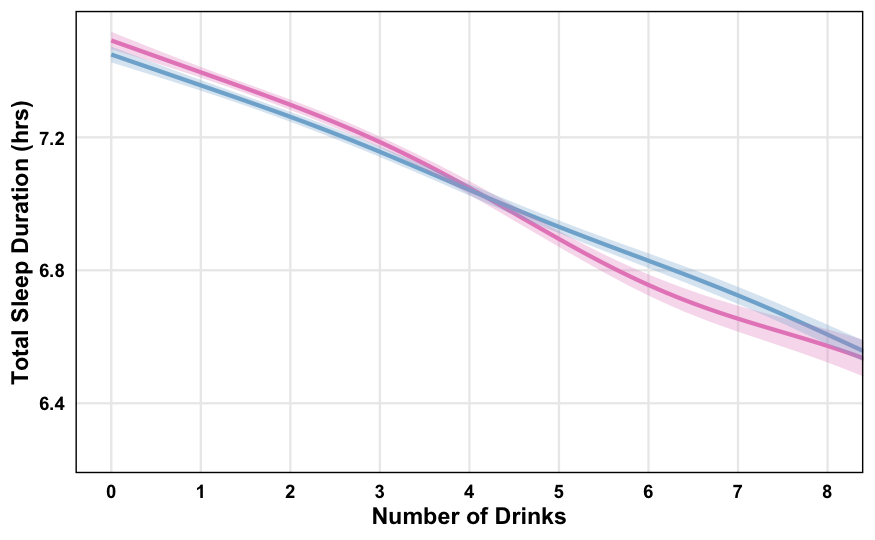

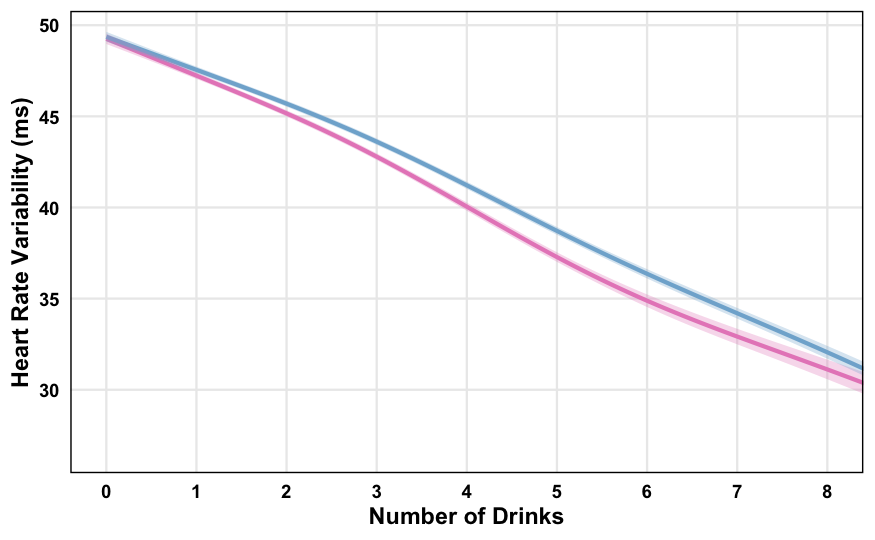

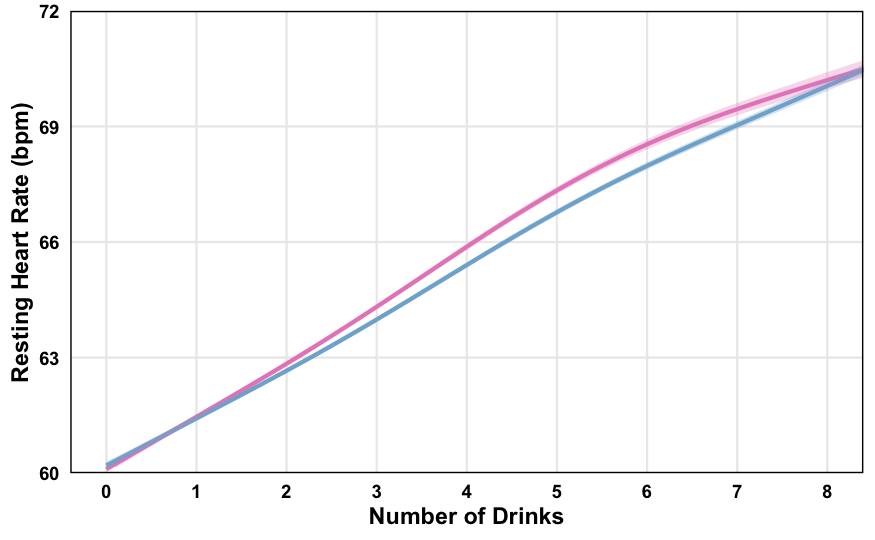

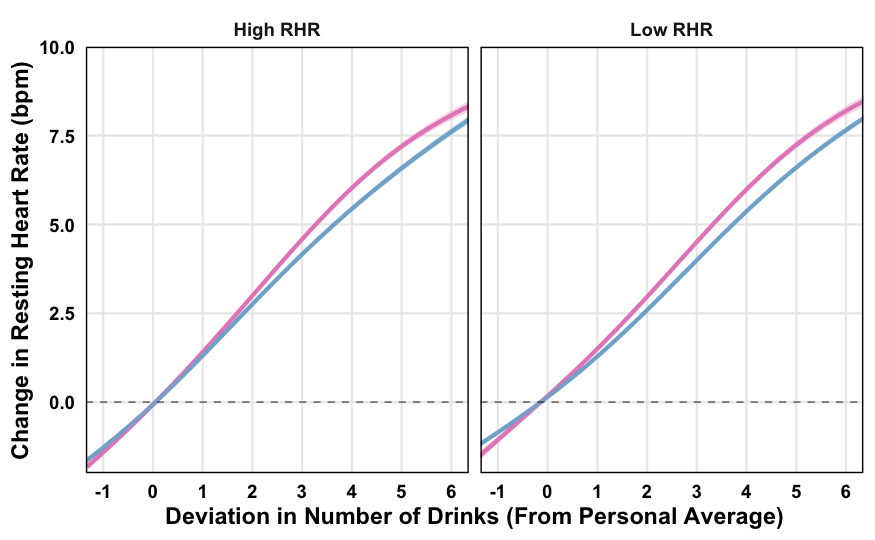

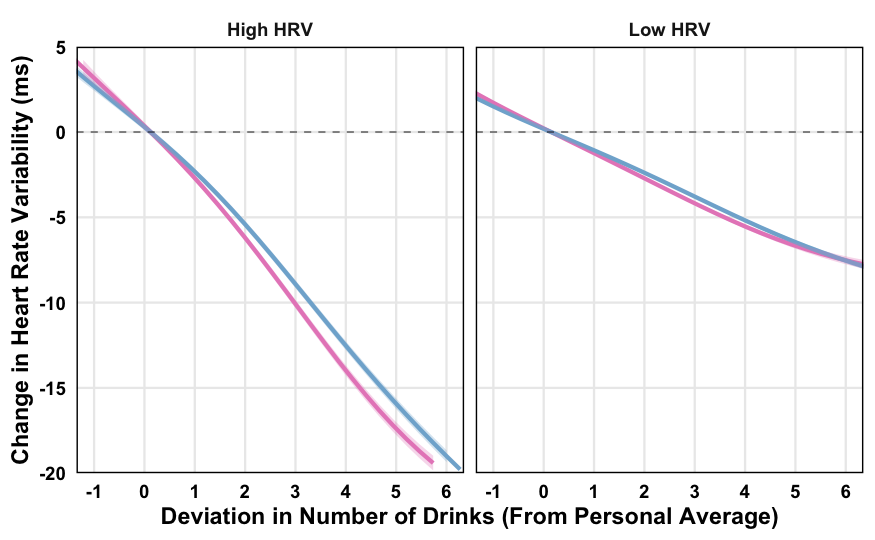

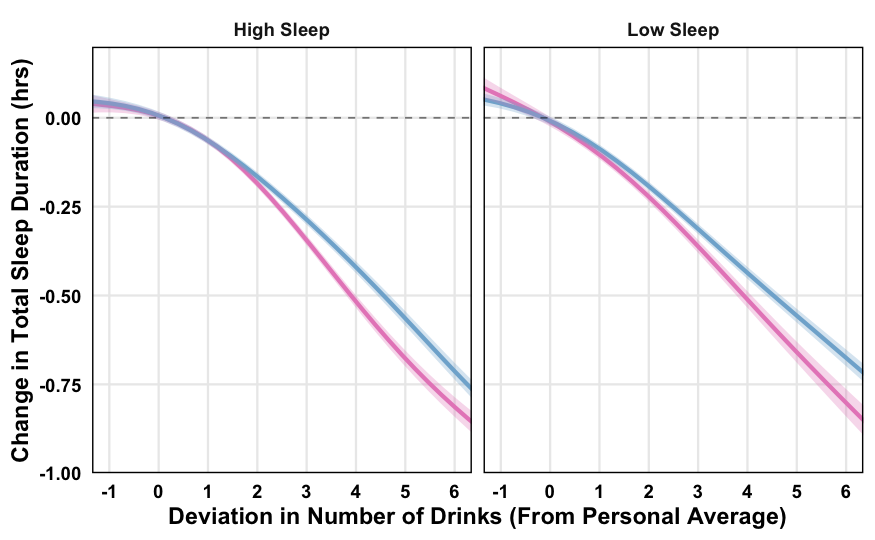

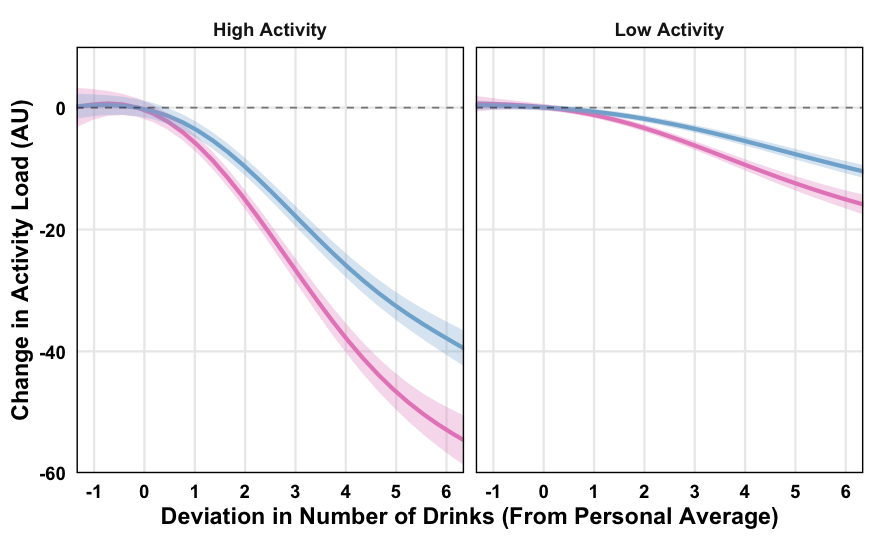


**Figure S2.** Sensitivity analysis examining whether biological sex-related differences in physiological and behavioral responses to alcohol vary by absolute drink amount. Generalized additive models estimated changes in resting heart rate (**A**), heart rate variability (**B**), sleep duration (**C**), and next-day physical activity (**D**), stratified by biological sex. Between-sex comparisons at specific drink quantities are shown in **S4 Table**.

**Biological Sex**


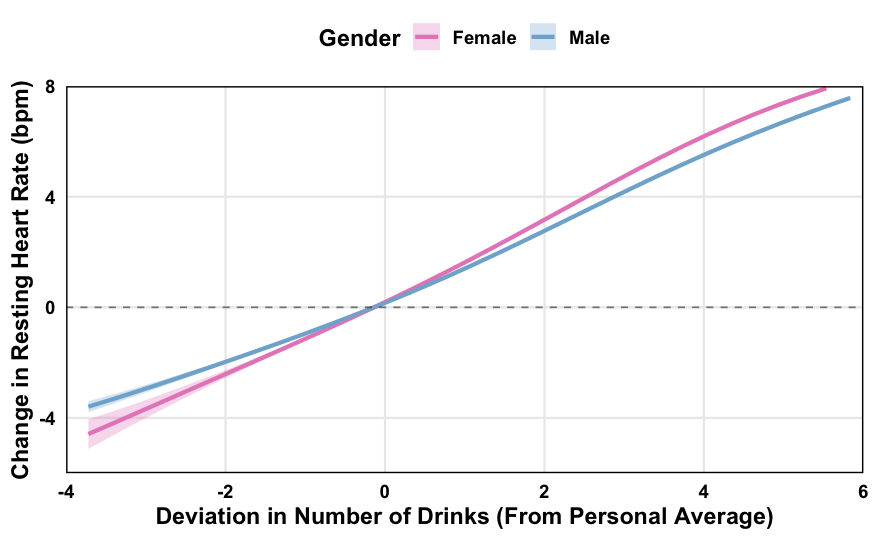


**A)**

**B)**

**C)**

**D)**
