# Supplementary material for: Real-world effects of alcohol on heart rate, sleep, and physical activity by age and sex
Source: PLOS Digit Health. 2026 Mar 9;5(3):e0001284. doi: 10.1371/journal.pdig.0001284 (PMC12970902; doi:10.1371/journal.pdig.0001284)

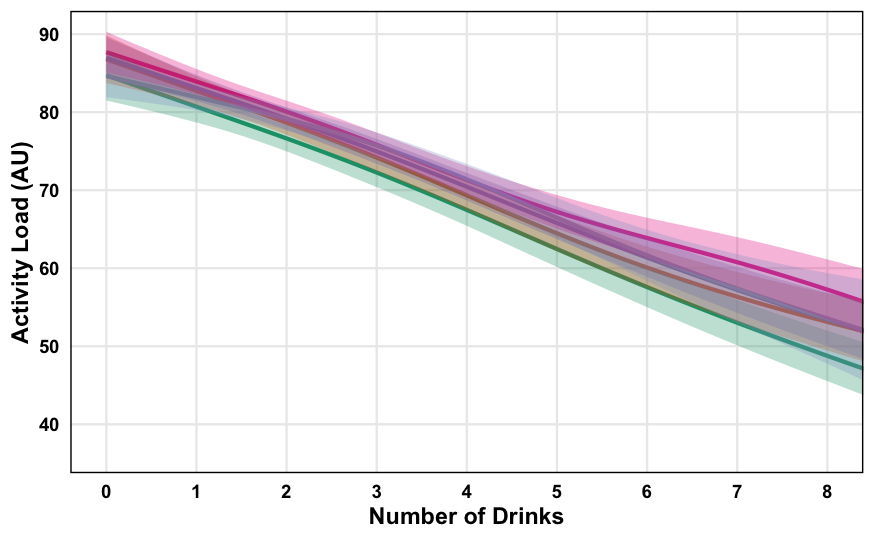

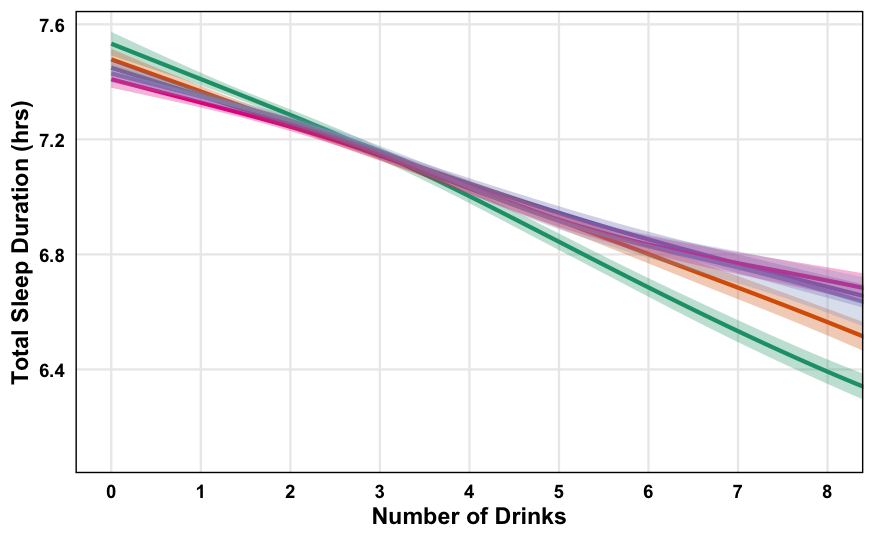

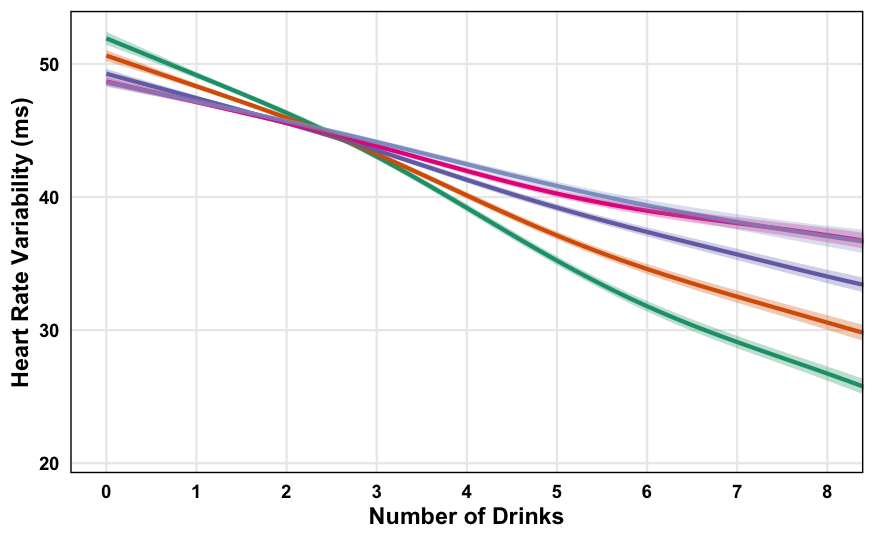

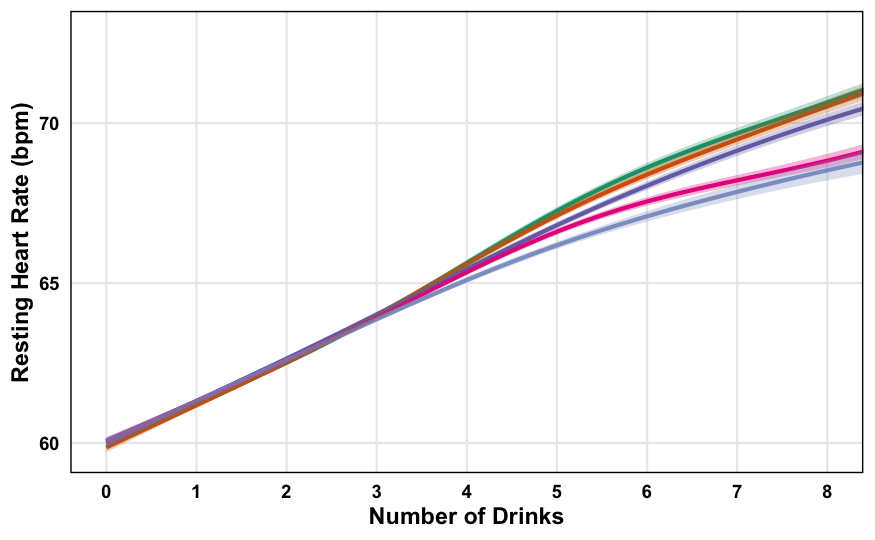

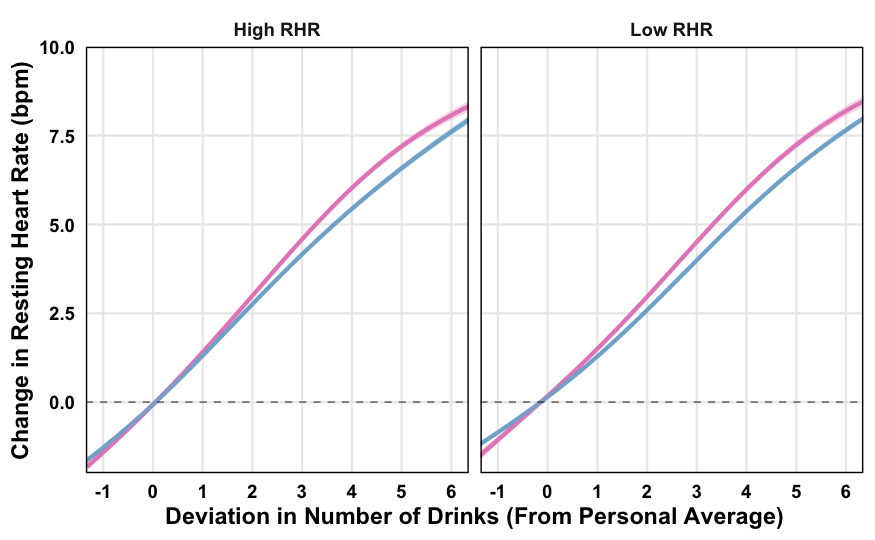

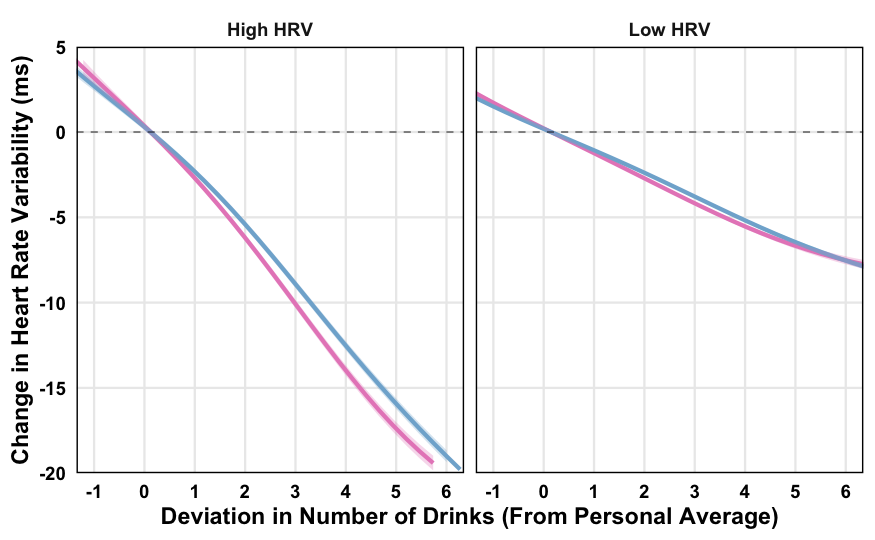

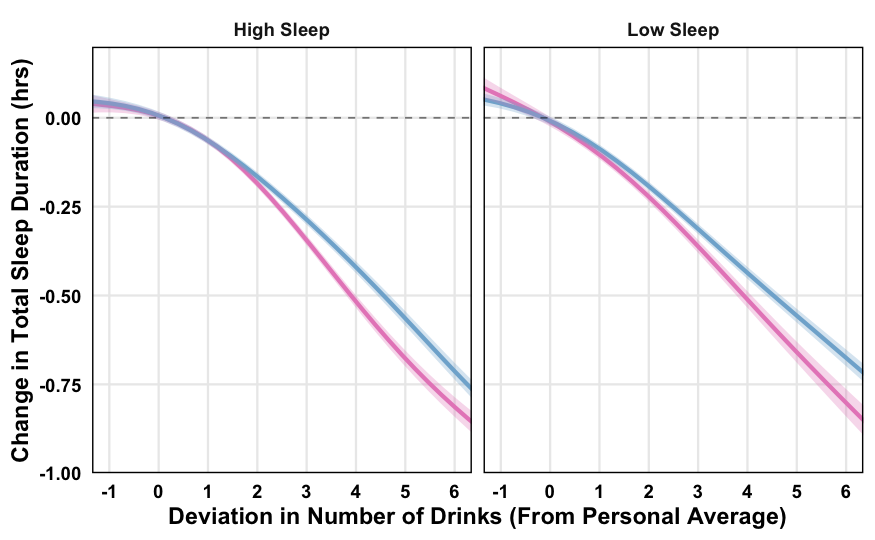

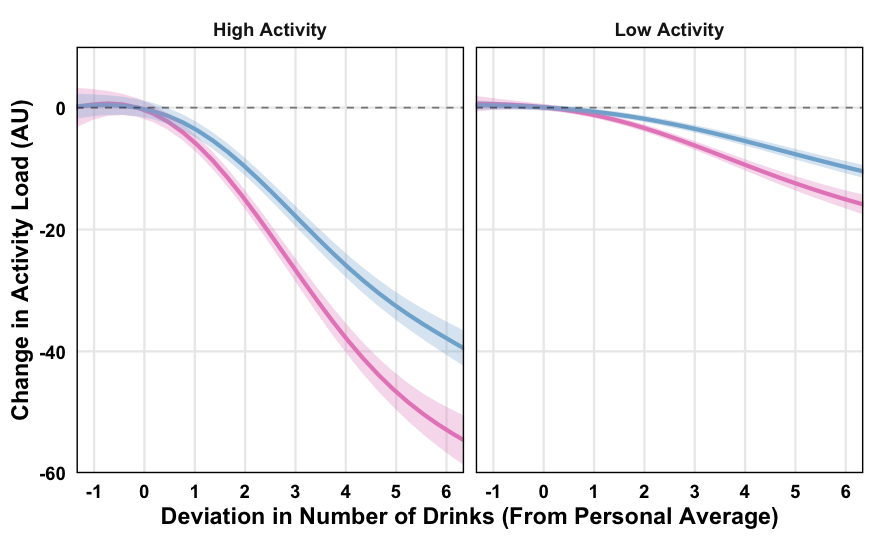


**Figure S4**. Sensitivity analysis examining whether age group-related differences in physiological and behavioral responses to alcohol vary by absolute drink amount. Generalized additive models estimated changes in resting heart rate (**A**), heart rate variability (**B**), sleep duration (**C**), and next-day activity (**D**), stratified by age group. Between age group comparisons at specific drink quantities are shown in **S9 Table**.

**D)**

**C)**

**B)**

**A)**

**Age Group**


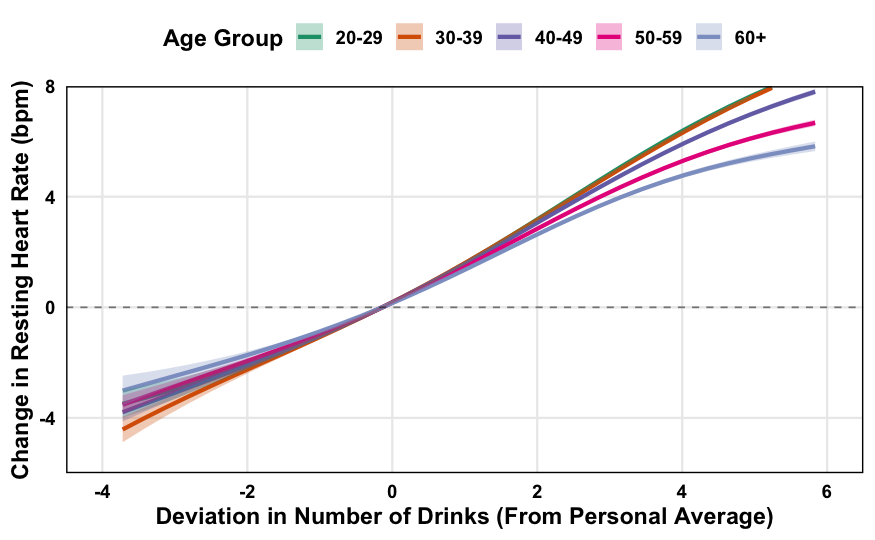

Supplement: S4 Fig — (DOCX) [file pdig.0001284.s018.docx]
