# Supplementary material for: Real-world effects of alcohol on heart rate, sleep, and physical activity by age and sex
Source: PLOS Digit Health. 2026 Mar 9;5(3):e0001284. doi: 10.1371/journal.pdig.0001284 (PMC12970902; doi:10.1371/journal.pdig.0001284)

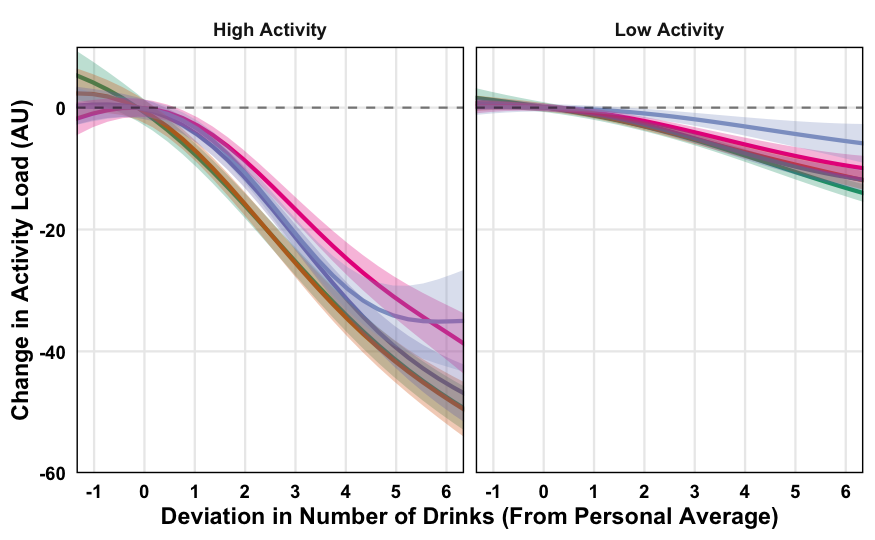

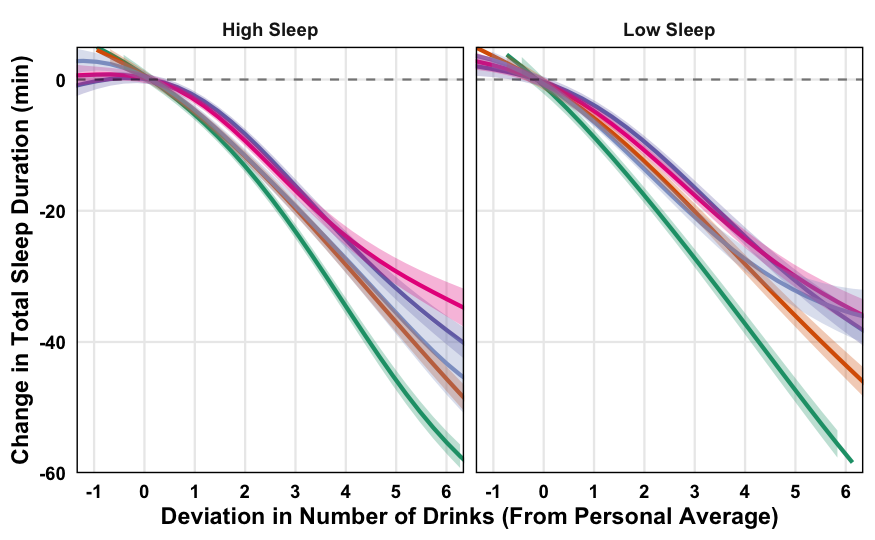

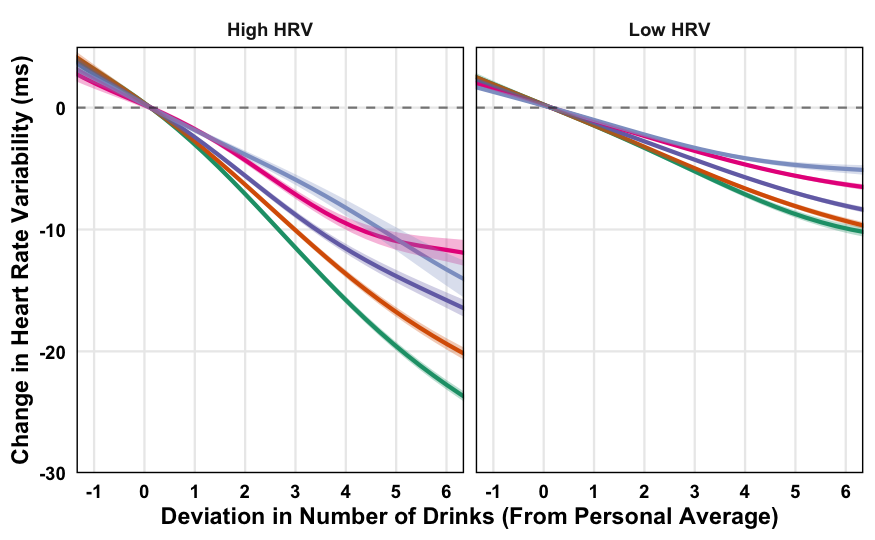

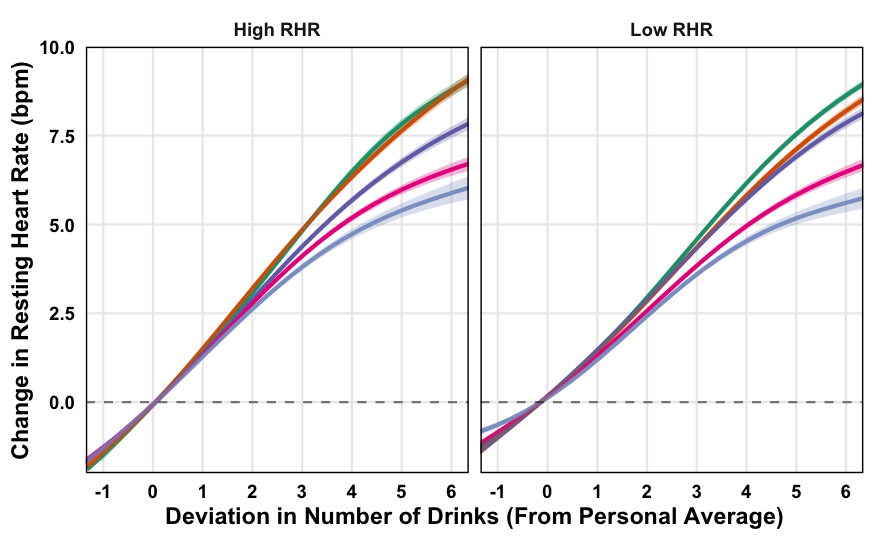

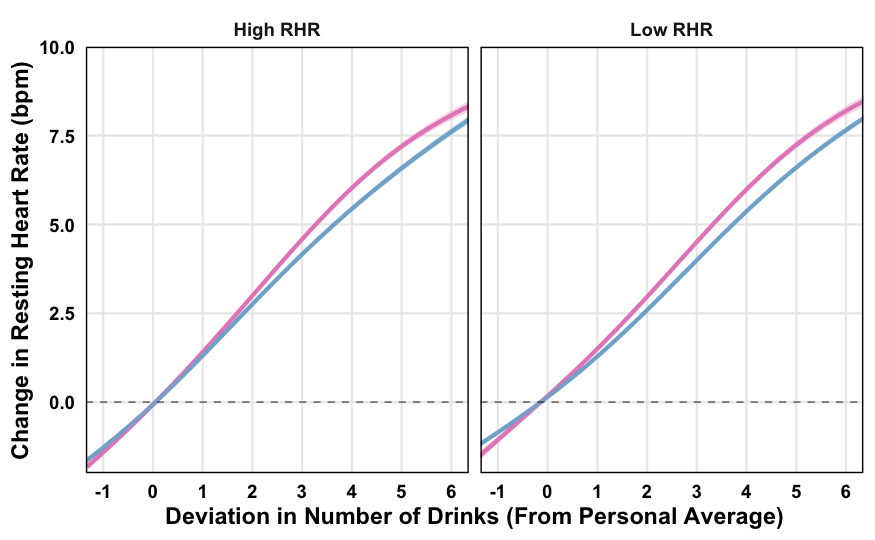

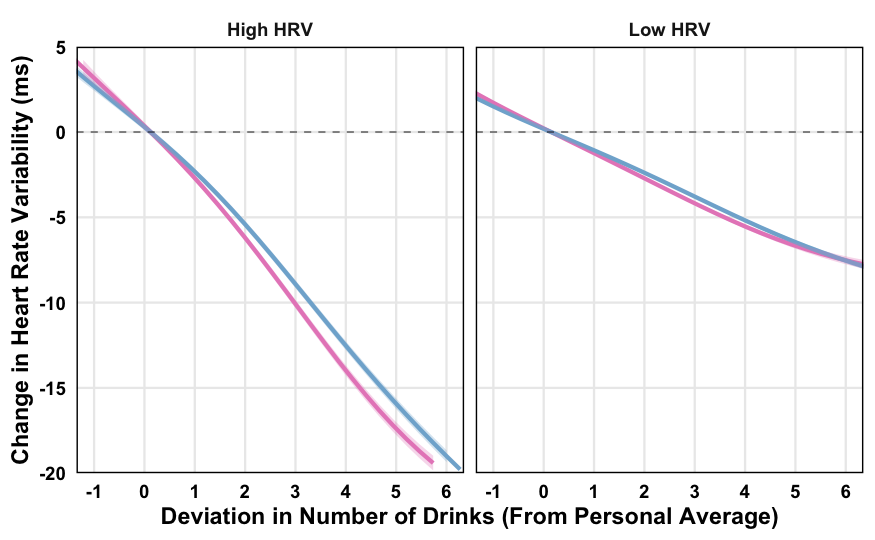

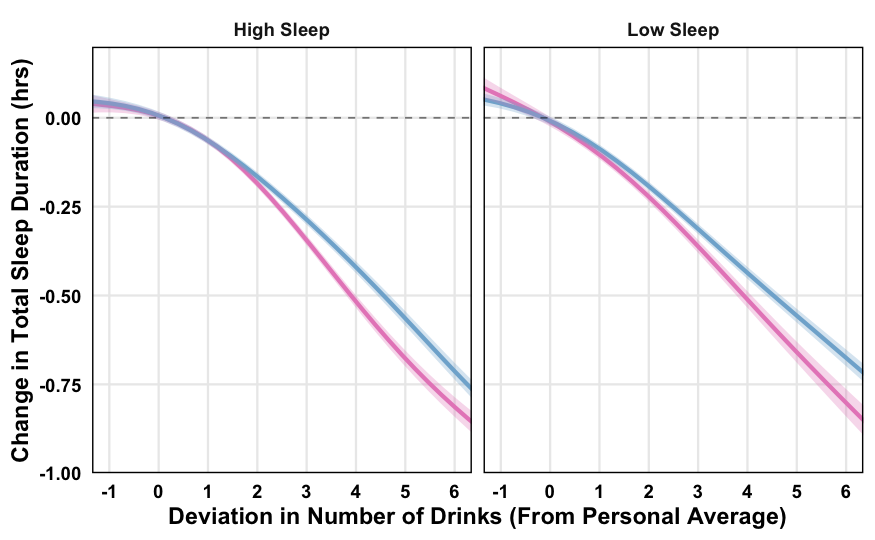

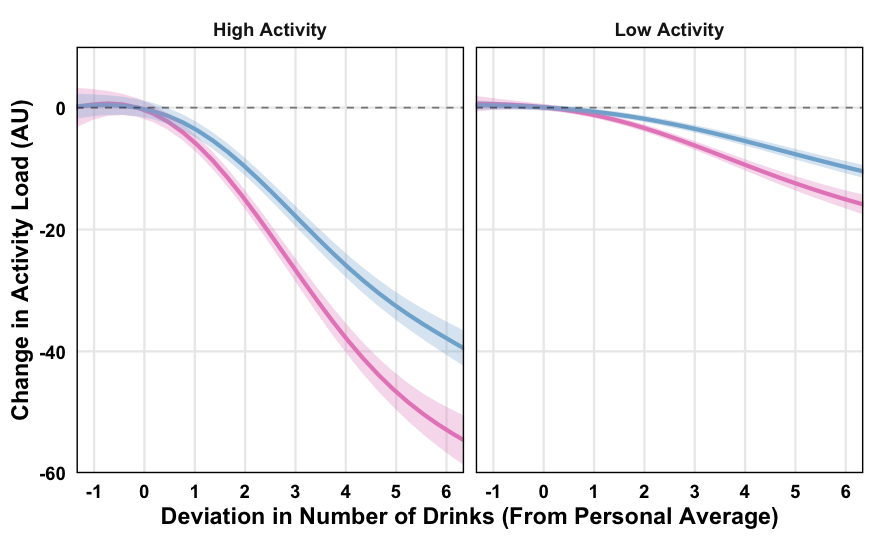


**Figure S5**. Sensitivity analysis examining whether age group-related differences in physiological and behavioral responses to alcohol vary by baseline physiology. Participants were split into high and low groups for resting heart rate (median = 59.77 bpm; **Panel A**), heart rate variability (median = 43.07 ms; **Panel B**), total sleep (median = 420 min; **Panel C**), and physical activity load (SHRZS; median = 65.15 AU; **Panel D**) based on median values of their respective person-level averages. Generalized additive models were then re-estimated separately with each group to assess changes in resting heart rate (**A**), heart rate variability (**B**), total sleep (**C**), and next-day activity (**D**) based on deviations in alcohol drink number.

**A)**

**B)**

**C)**

**D)**

**Age Group**


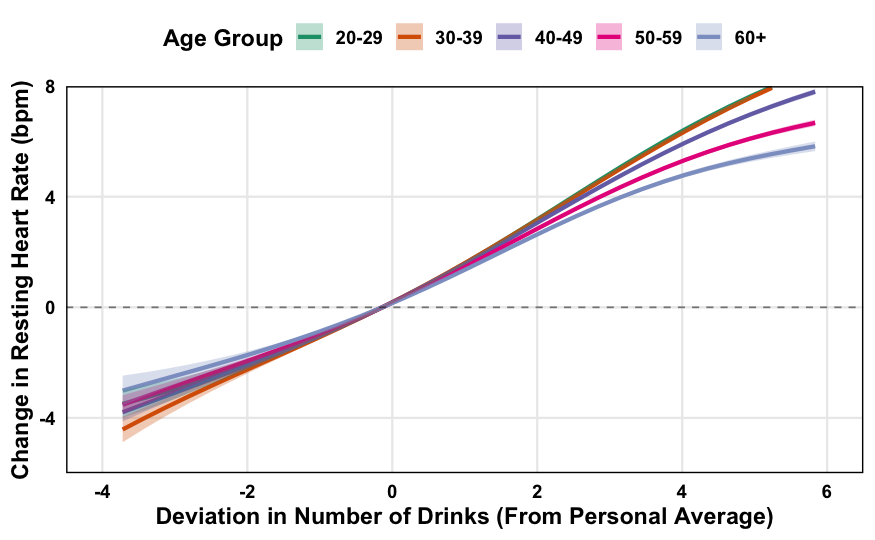

Supplement: S5 Fig — (DOCX) [file pdig.0001284.s019.docx]
