# Supplementary material for: Real-world effects of alcohol on heart rate, sleep, and physical activity by age and sex
Source: PLOS Digit Health. 2026 Mar 9;5(3):e0001284. doi: 10.1371/journal.pdig.0001284 (PMC12970902; doi:10.1371/journal.pdig.0001284)
Supplement: S6 Fig — (DOCX) [file pdig.0001284.s020.docx]

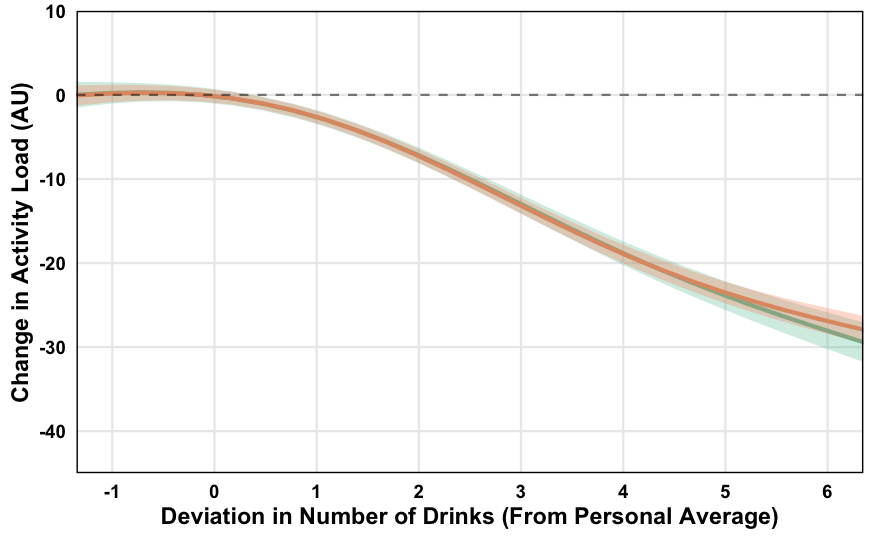

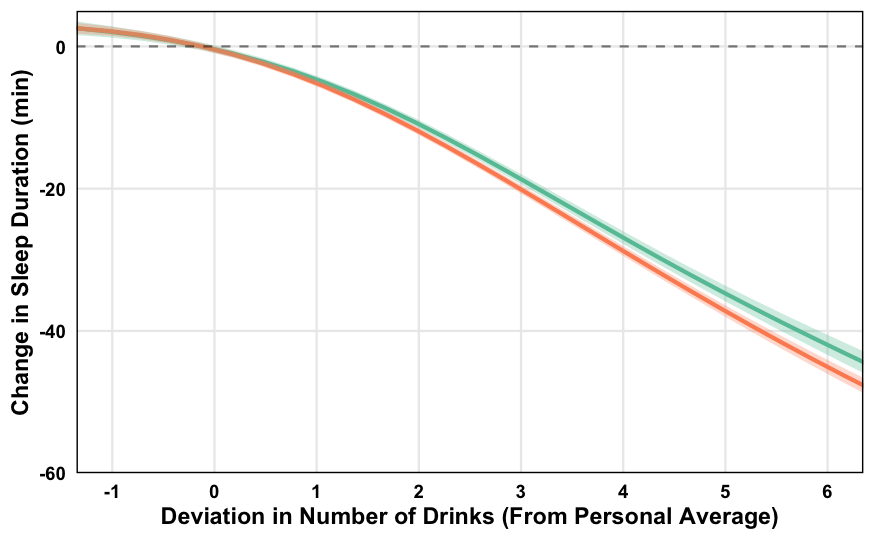

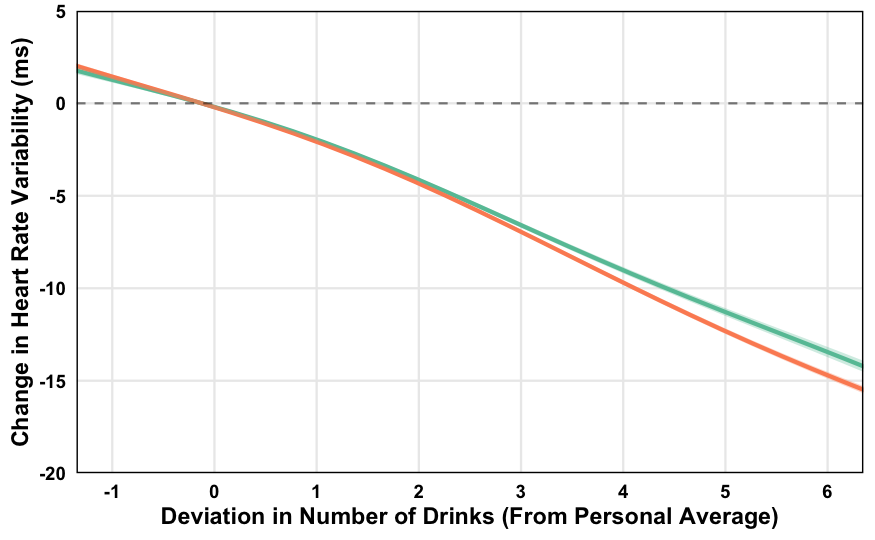

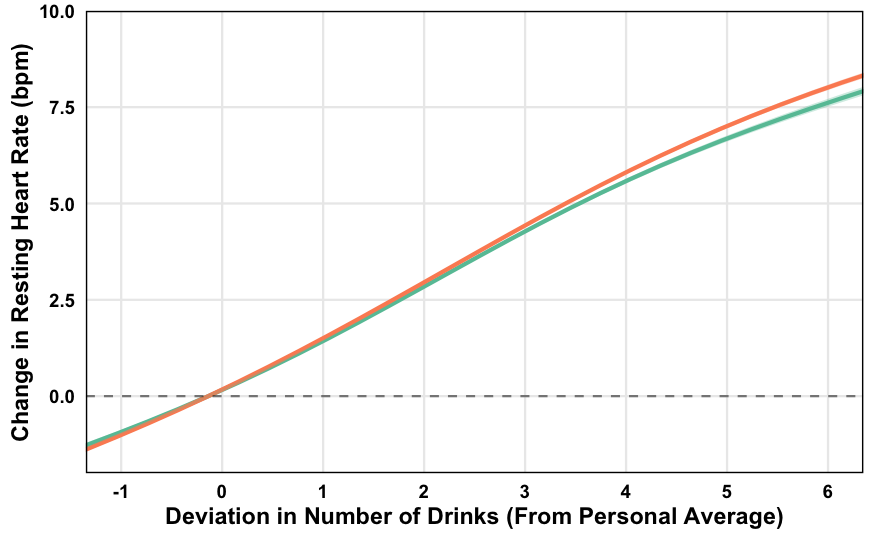

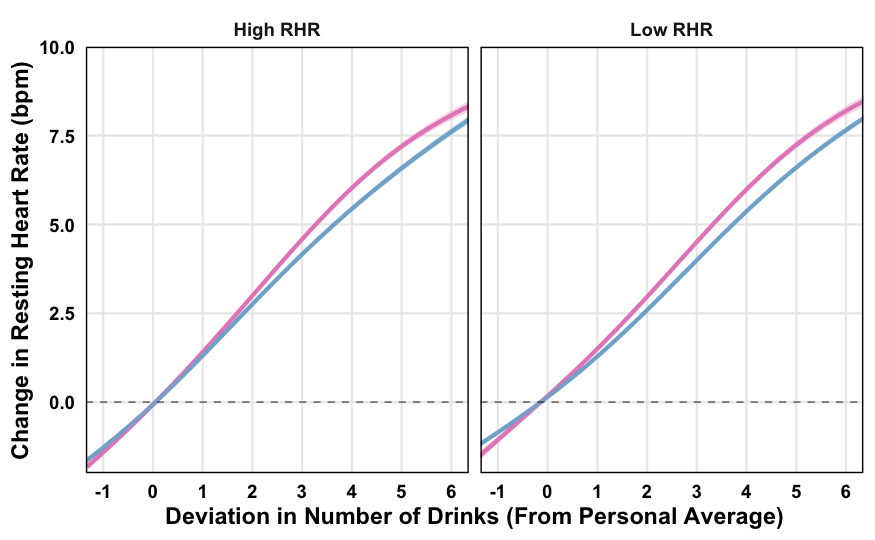

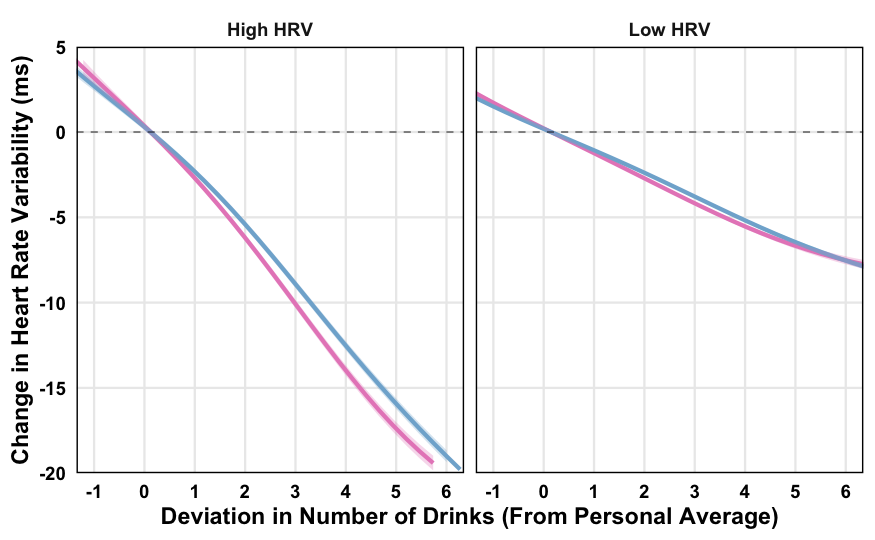

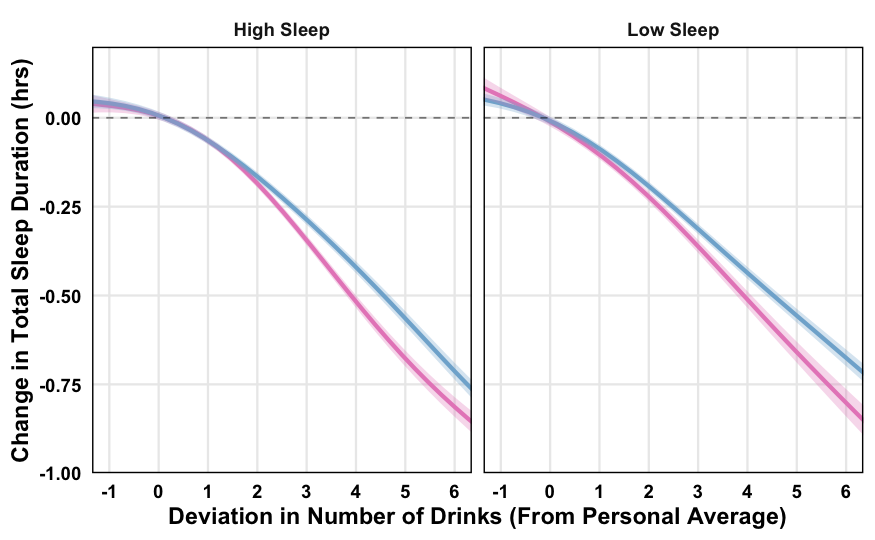

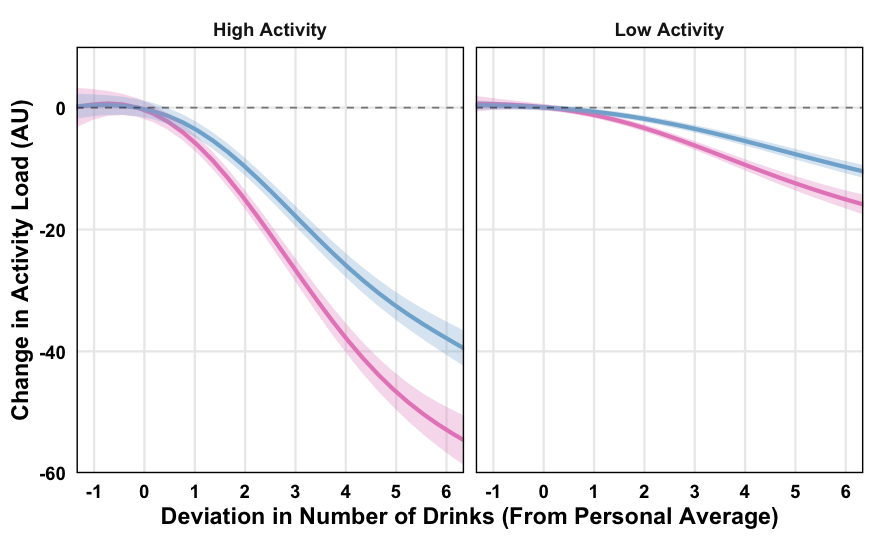


**Figure S6.** Moderation of within-person associations between deviation in alcoholic drink number and physiological and behavioral outcomes by self-reported hydration status. Generalized additive models estimated changes in resting heart rate (**A**), heart rate variability (**B**), sleep duration (**C**), and next-day activity (**D**) based on deviations from individuals’ personal average number of drinks. Nights were stratified by self-reported hydration status (True = 1,747,958 days/nights vs. False = 3,361,227 days/nights), with “True” indicating participants reported hydration in their journal. Contrasts between hydration groups at different drink quantities are presented in **S14 Table**.

**Hydration**


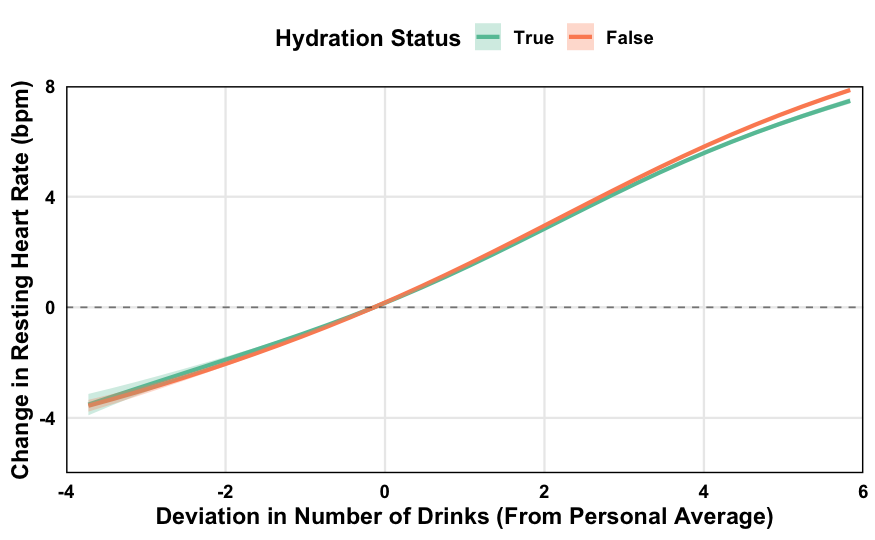


**A)**

**B)**

**C)**

**D)**
