# Supplementary material for: Real-world effects of alcohol on heart rate, sleep, and physical activity by age and sex
Source: PLOS Digit Health. 2026 Mar 9;5(3):e0001284. doi: 10.1371/journal.pdig.0001284 (PMC12970902; doi:10.1371/journal.pdig.0001284)
Supplement: Attachment — Submitted filename: Alcohol_PlosDH_ReviewerResponse_R1_07_Jan_2026.docx [file pdig.0001284.s022.docx]

Dear Dr. Celi and the Reviewers,

Thank you for the thorough and constructive feedback on our manuscript. We have revised the manuscript extensively in response to the reviewers’ comments and believe it is substantially stronger as a result.

We are grateful for the opportunity to revise and resubmit our manuscript, and appreciate your time and effort in reviewing. For convenience, reviewer comments are provided in black font, and **our responses in blue and bolded**. Wherever possible, we have included updated text from the manuscript *in italics* to streamline your review process.

**Editor**

This manuscript presents an impressive analysis of over 5 million person-days from nearly 21,000 participants who used the WHOOP Strap 4.0 wearable device, examining the acute physiological and behavioural effects of alcohol consumption. The study's considerable strengths lie in its unprecedented scale for digital health research, its within-person design that controls for individual variability, and its systematic examination of how age and biological sex moderate alcohol's effects on cardiovascular measures, sleep, and physical activity. The finding that females and younger adults experience more pronounced disruptions is particularly noteworthy and contributes meaningfully to our understanding of individual differences in alcohol response. The reviewers appreciate the practical insights regarding behavioural modifications that may attenuate alcohol's adverse effects, such as earlier drinking timing and prioritising adequate sleep, which have clear relevance for both individual health behaviours and population-level public health messaging. However, the reviewers raised several substantive methodological and reporting concerns that require careful attention before the manuscript can be recommended for publication.

**Thank you for your thoughtful evaluation of our manuscript and for highlighting both its strengths and areas requiring revision or clarification. We appreciate the time and care dedicated to the review process. We have revised the manuscript extensively and addressed each of the issues raised below on a point-by-point basis. We hope the changes and additions satisfactorily resolve the concerns and strengthen the paper for publication.**

First, the conflict of interest warrants more substantive discussion. Seven of the eleven authors, including the first and senior authors, are employees of WHOOP, Inc., the manufacturer of the device used in this study. While disclosed, the statement that "WHOOP has no stake in the outcome of this research" inadequately acknowledges that research demonstrating a commercial device's sensitivity to lifestyle factors inherently promotes the device's utility and market value. The limitations section should more transparently address how this conflict was managed and its potential implications for data interpretation.

**We appreciate this point and are happy to provide a more transparent acknowledgement and clarification within the manuscript. We have made two key revisions.**

1. **Revised Competing Interests Statement (lines 32-39): “***The study was funded by WHOOP, Inc. GJG, WvH, DMP, FF, JK, CC, and KEH are employees of WHOOP, Inc., and GJG, DMP, FF, JK, CC, and KEH hold stock options as part of their employee compensation, which represents a potential financial interest. These authors contributed to the study design, data curation, statistical analysis, and manuscript preparation. All analyses followed a prespecified workflow, and analytic decisions were made solely within the research and data science teams. Marketing, enterprise, and other non-research groups at WHOOP, Inc. had no role in study approval, data interpretation, manuscript preparation, or decisions regarding submission or publication. The remaining authors declare no competing interests*.”
2. **Added to the limitations (lines 242-249): “***An additional consideration relates to the conflict of interest. Several authors are employees of WHOOP, Inc., some of whom hold stock options, and the research was funded by WHOOP, Inc. While the analyses were conducted independently by the research and data science teams and no commercial divisions were involved, it is important to acknowledge that demonstrating sensitivity of physiological measures to behavioral exposures, such as alcohol could have favorable commercial ramifications for wearable devices and perhaps WHOOP in particular. Readers should therefore interpret these findings in this context, although the methodological transparency and prespecified analytical workflow help mitigate concerns regarding undue influence.*”

Second, the reviewers identified critical statistical issues that must be addressed. Given the very large sample size, statistical significance testing yields extremely small p-values for virtually all comparisons, including those in the fourteen supplementary tables. The manuscript needs to shift emphasis from statistical significance to effect sizes and clinical or practical significance, providing readers with clear guidance on which observed differences are likely to be meaningful in real-world contexts. More problematically, the study reports results from 16 primary models plus extensive supplementary analyses without applying appropriate corrections for multiple comparisons. While the authors set alpha at 0.005 rather than 0.05, this does not address the family-wise error rate inflation from testing multiple hypotheses. The reviewers questioned why Bonferroni or similar corrections were not applied across the model family, and why all hypothesis tests in Tables S1-S14 are not factored into a comprehensive multiple comparison correction. This is a fundamental statistical concern that undermines confidence in which findings represent true effects versus Type I errors.

**We appreciate the emphasis on practical and clinical interpretation in the light of the very large sample size. We agree that statistical significance alone is not sufficient for conveying meaningful effects, and that multiple hypothesis testing must be addressed. Accordingly, we have made several revisions to strengthen rigor, clarify magnitude, and reduce concerns regarding family-wise error inflation:**

1) **Added standardized marginal effect sizes (ES) for all contrasts. The ES is now reported for every contrast in the Results and Supplemental Tables to contextualize the magnitude and relevance of each effect.** **This is now included in our Statistical Analysis section (line 342-343): “***Contrast estimates were standardized by dividing by the model residual standard deviation, yielding standardized effect sizes (ES).*”
2) **Tightened the alpha to 0.001 for all sixteen primary models, which is more conservative than a Bonferroni correction for 16 tests (0.05 / 16 = 0.003) (lines 353-355): “***To conservatively account for 16 primary models, statistical significance was set at α=0.001, which is stricter than a Bonferroni correction of 0.003. Accordingly, 99.9% confidence intervals are reported.*”
3) **Clarified in the Statistical Analysis that all pairwise comparisons already used a multivariate t-distribution, which controls for Type 1 error within each model family (lines 341-342): “***All pairwise comparisons used the multivariate t-distribution to control for family-wise error rate.*”
4) **Revised Results to emphasize the magnitude of effects. For example (lines 123-129): “***For example, consuming one drink more than usual (vs. one less) was associated with an increase in RHR by 2.8 bpm (99.9% CI: 2.7, 2.9) in females and 2.4 bpm (99.9% CI: 2.3, 2.4) in males, corresponding to moderate standardized effects (ES=0.61 in females and ES=0.52 in males). HRV concurrently declined by 3.8 ms (99.9% CI: -4.1, -3.5) in females and 3.3 ms (99.9% CI: -3.5, -3.1) in males, reflecting small effect sizes (ES=0.30 in females and ES=0.26 in males). These effects scaled with increasing alcohol intake. Consuming five drinks more than usual (vs. three more) led to HRV reductions of 5.6 ms (99.9% CI: -5.9, -5.3) in females and 5.1 ms (99.9% CI: -5.3, -4.9) in males (ES=0.45 in females and ES=0.41 in males).”*
5) **Acknowledged small effect magnitudes explicitly in the Limitations (lines 237-239) “***Fourth, many estimated effects were small in magnitude, consistent with acute physiological responses in real-world conditions. However, sequential dose-response contrasts reveal that small stepwise changes compound at higher intake levels.*”
6) **To support analytic transparency and demonstrate that analyses were hypothesis-driven, the preregistered Statistical Analysis Plan (time-stamped prior to analysis; April 21, 2025) is available for review. This document specifies the primary models, contrasts, and analytical workflow (lines 368-369): “***The preregistered statistical analysis plan is archived at Open Science Framework (*[*https://osf.io/zf5sp*](https://osf.io/zf5sp)*).*”

Third, several methodological choices require better justification or revision.

The timing of sampling (after device activation but before checking eligibility criteria) is inefficient and should be clarified.

**We appreciate the request for clarification regarding the order of sampling and eligibility screening. Sampling was performed prior to eligibility filtering to reduce computational burden, as querying full multi-million row wearable and journal tables for all active users would have required extracting far more data than were ultimately analyzed. Eligibility criteria were then applied after sampling. We present the full filtering sequence in Figure 1 for transparency.**

The decision to model sleep and physical activity moderators separately rather than jointly limits understanding of their potential interactions.

**We appreciate this point. Our moderation models were specified a prior in the Statistical Analysis Plan (Aim 3), which focused on two questions:**

1) **Whether sleeping more (or less) than usual after drinking modified the effects of drinking on RHR, HRV, and next-day activity, and**
2) **Whether exercising more (or less) than usual on the day of drinking modified the effects of drinking on RHR, HRV, and sleep that night.**

**Because these questions were specified independently, sleep and activity moderators were modeled separately in the primary analysis and were not jointly included in the model.**

**We agree that jointly estimating moderation could provide additional insight. In response to the editor’s suggestion, we conducted an additional analysis that included both sleep and activity simultaneously. In these models, the nonlinear interactions between sleep and activity with drinks remained virtually unchanged. Both the shape and the magnitude of effects closely matched the preregistered model.**

**Consistent with the preregistered analysis plan and to preserve interpretability, our primary results continue to report one moderator at a time. However, the additional joint-moderation analysis provides reassurance that our conclusions are robust to the inclusion of both behavioral factors. We have added text to the Statistical Analysis section to clarify this rationale (lines 348-350): “***Moderators were modeled separately to align with the preregistered analysis plan and to maintain interpretability. Exploratory analysis including both sleep and activity together yielded similar results, supporting the robustness of the primary findings.”*

The choice of person-mean centering, while reasonable, has implications for interpretation that should be acknowledged in the limitations.

**We agree that our use of person-mean centering has important implications for interpretation. We have revised the limitations section to explicitly note that this analytic approach focuses on within-person associations and thereby supports inference about acute, short-term deviations rather than between-person differences or long-term health implications (lines 239-242): “***Fifth, we focused on acute physiological and behavioral consequences of alcohol using a within-person (via person-mean centering) framework. Accordingly, findings reflect short-term deviations from an individual’s typical physiology and behavior rather than between-person differences or long-term health implications.*”

The composite physical activity score, while previously used by this research group, would benefit from additional supporting references to establish its broader validity.

**We have strengthened the description and justification of the composite physical activity metric (lines 293-304): “***Daily physical activity was calculated using the summated-heart-rate-zone score (SHRZS), a validated measure of internal training load developed by Edwards (29) and widely used across athletic and occupational populations (30-33). Each participant’s maximum heart rate was estimated using the non-linear formula 192–0·007*age^^2^ (34), manually set, or defined as their highest recorded heart rate. Physical activity was either detected automatically by the WHOOP analytics platform (~85% of cases) or logged manually by the participant to capture activities not automatically detected by the device. Importantly, regardless of detection method, activity intensity was derived from continuous heart rate data rather than manual input. Continuous heart rate data during each activity was used to calculate time spent in five zones defined as percentages of maximum heart rate: zone 1 (50-60%), zone 2 (60-70%), zone 3 (70-80%), zone 4 (80-90%), and zone 5 (90-100%). Minutes in each zone were multiplied by zone-specific weights (i.e., zone 1=1, zone 2=2, etc.) and summed to yield the SHRZS. This approach has demonstrated strong associations with session rating of perceived exertion and other internal and external load metrics across diverse populations (30-33).*”

Fourth, important measurement and data quality issues need clarification.

The manuscript does not describe how artifacts were handled in the RMSSD calculation, despite HRV's known sensitivity to measurement artifacts.

**We have clarified the artifact-handling procedure used in the derivation of nocturnal HRV. The Methods section has been revised to specify that (lines 284-292): “***RHR and HRV were derived from nocturnal sleep using a weighted average approach, prioritizing more stable periods of slow-wave sleep (28). RHR was defined as the average number of heart beats per minute during the primary sleep episode. HRV was calculated as the root mean squared of successive beat-to-beat interval differences (RMSSD). To mitigate the influence of motion and signal artifacts, epochs identified as wake or as having low signal quality were excluded prior to aggregation. Remaining epochs were combined using a weighted average, with higher weights assigned to epochs with higher likelihood of slow-wave sleep and those occurring later in the sleep period. Sleep duration was defined as total sleep time. WHOOP (WHOOP, Inc. Boston, MA) has been validated against electrocardiography and polysomnography for heart rate (99% agreement) and 2-stage sleep categorization (86-89% agreement) (23, 24).*

The protocol for reconciling manual physical activity entries with automated detection requires explanation.

**We have clarified this point in the Methods section. Manually activity logging is used to identify activities not automatically detected by the device, whereas activity intensity is derived from continuous heart rate data during those periods of time. Participants cannot manually input heart rate zones or intensity. As such, the summated heart-rate-zone score reflects measured internal load regardless of whether an activity was detected automatically or logged manually. This clarification has been added to the Methods (lines 293-304): “***Daily physical activity was calculated using the summated-heart-rate-zone score (SHRZS), a validated measure of internal training load developed by Edwards (29) and widely used across athletic and occupational populations (30-33). Each participant’s maximum heart rate was estimated using the non-linear formula 192–0·007*age^^2^ (34), manually set, or defined as their highest recorded heart rate. Physical activity was either detected automatically by the WHOOP analytics platform (~85% of cases) or logged manually by the participant to capture activities not automatically detected by the device. Importantly, regardless of detection method, activity intensity was derived from continuous heart rate data rather than manual input.*”

The limitations should acknowledge recall bias inherent in manual alcohol consumption logging.

**We have clarified this point in the Limitations section. The text now explicitly notes that alcohol intake was self-reported and therefore subject to recall bias, in addition to uncertainty in dose and timing (lines 230-231): “***First, alcohol intake was self-reported in a real-world setting, which introduces recall bias as well as uncertainty in dose and timing.*”

Table 1 reports numerous statistically significant comparisons despite very large standard deviations, which appears inconsistent and likely reflects the enormous sample size rather than meaningful differences; this reinforces the need to emphasize effect sizes throughout.

**We agree with this assessment. In revisiting Table 1, we identified several variables were not normally distributed. Normality was assessed using the Shapiro-Wilk test and Q-Q plots, and summary statistics were revised accordingly. Table 1 now presents normally distributed variables as mean ± standard deviation and non-normally distributed variables as median (interquartile range). The table legend has been updated to clarify this distinction.**

**More broadly, we agree that the statistical significance in large samples can be obscure practical relevance. As described in the revised Statistical Analysis section, we have tightened the significance threshold (α=0.001) and reported complementary** **standardized effect sizes (ES) for all contrasts to contextualize the magnitude and relevance of observed effects.**

The manuscript addresses an important question using an impressively large dataset and rigorous within-person analytical framework. The findings regarding sex and age differences in alcohol's acute physiological effects, along with the examination of behavioural moderators, represent genuine contributions to the digital health literature. However, the statistical issues regarding multiple comparisons, the inadequate discussion of conflicts of interest, and the various methodological clarifications required are substantial concerns that must be satisfactorily addressed before the manuscript can be considered suitable for publication in PLOS Digital Health. With appropriate revisions, particularly regarding the multiple comparisons problem and the emphasis on effect magnitude over statistical significance, this work has the potential to make a valuable contribution to understanding alcohol's real-world physiological impacts through wearable technology.

**We thank the Editor for the careful, thoughtful, and constructive evaluation of our manuscript. We are grateful for the opportunity to revise and resubmit our work, and we appreciate the time and effort devoted to the review process. We believe that the revisions made in response to these comments have substantially strengthened the manuscript, particularly with respect to methodological transparency, statistical rigor, and interpretability. We have addressed each concern in detail above (and below) and hope that the changes satisfactorily resolve the issues raised. We look forward to your consideration and hope the revised version is now suitable for publication in PLOS Digital Health.**

**Reviewer 1**

The manuscript presents a robust analysis of a very large, real-world dataset from a commercial wearable device to quantify the acute effects of alcohol use on nocturnal cardiovascular measures (RHR, HRV), sleep, and next-day activity levels. A key strength is the within-person design, analyzing over 5 million person-days from nearly 21,000 participants to isolate the effects of alcohol relative to a user's own baseline. The study demonstrates that alcohol increases RHR, decreases HRV, and modestly reduces sleep and activity, with a novel finding that these effects are more pronounced in females and younger adults. The investigation into some of the “mitigating” factors (e.g., drink timing, sleep duration) provides some practical insights.

**We thank the reviewer for the thoughtful and encouraging evaluation of our work. We appreciate the recognition of the study’s scale, within-person design, and the practical relevance of the findings. We believe the revisions made in response have strengthened the manuscript.**

Major Comment: The study's funding and the employment of 7 of 11 authors (including the first and senior authors) by the device manufacturer (WHOOP, Inc.) represent a significant conflict of interest. While disclosed, this COI could be more substantially addressed in the limitations. The current disclosure states "WHOOP has no stake in the outcome of this research", which is debatable, as findings that highlight the device's sensitivity to lifestyle factors (like alcohol) inherently promote the device's utility and value.

**We thank the reviewer for raising this point. We agree that the potential conflict of interest warrants closer and more transparent acknowledgement. In response we have revised both the Competing Interests Statement and the limitations section to more explicitly detail the nature of the financial relations, how potential conflicts were managed, and how readers should interpret findings in this context.**

1. **Competing Interests Statement (lines 32-39): “***The study was funded by WHOOP, Inc. GJG, WvH, DMP, FF, JK, CC, and KEH are employees of WHOOP, Inc., and GJG, DMP, FF, JK, CC, and KEH hold stock options as part of their employee compensation, which represents a potential financial interest. These authors contributed to the study design, data curation, statistical analysis, and manuscript preparation. All analyses followed a prespecified workflow, and analytic decisions were made solely within the research and data science teams. Marketing, enterprise, and other non-research groups at WHOOP, Inc. had no role in study approval, data interpretation, manuscript preparation, or decisions regarding submission or publication. The remaining authors declare no competing interests*.”
2. **Limitations (lines 242-249): “***An additional consideration relates to the conflict of interest. Several authors are employees of WHOOP, Inc., some of whom hold stock options, and the research was funded by WHOOP, Inc. While the analyses were conducted independently by the research and data science teams and no commercial divisions were involved, it is important to acknowledge that demonstrating sensitivity of physiological measures to behavioral exposures, such as alcohol could have favorable commercial ramifications for wearable devices and perhaps WHOOP in particular. Readers should therefore interpret these findings in this context, although the methodological transparency and prespecified analytical workflow help mitigate concerns regarding undue influence.*”

Minor Comments
Given the very large N, nearly all tests are statistically significant. Please ensure that the text (stats, and especially in the Discussion) focuses on the magnitude and clinical significance of these effects.

**We agree with this concern and appreciate the reviewer’s emphasis on interpretability. In response, we have revised the manuscript to complement hypothesis testing with measures of effect size magnitude. Specifically, we now report standardized effect sizes (ES) for all contrasts, tightened the significance threshold to α=0.001, and revised the Results and Discussion sections to emphasize the magnitude and dose-response nature of observed effects. We also explicitly acknowledge the generally small effect sizes and their interpretation in the context of acute, real-world physiological and behavioral responses in the Discussion section. The six specific changes made are described in greater detail above in our second response to the Editor.**

Please consider placing the METHODS section after the Introduction, as is typical of most major journals.

**We appreciate the suggestion, however, PLOS Digital Health specifies a manuscript structure in which the Materials and Methods section follows the Discussion. We have therefore retained the journal-recommended organization to ensure consistency with PLOS formatting guidelines:** [**https://journals.plos.org/digitalhealth/s/submission-guidelines**](https://journals.plos.org/digitalhealth/s/submission-guidelines)**.**

**Reviewer 2**

Line 62 - I suggest using ":" instead of ". For example,"

**We have revised the sentence for clarity per the reviewer’s recommendation (lines 61-65): “***These changes were more pronounced in females than males and in younger than older adults: consuming one drink more than personal average, compared with one less, was associated with an increase in RHR by 2.8 bpm (99.9% CI: 2.7, 2.9) in females and 2.4 bpm (99.9% CI: 2.3, 2.4) in males, while HRV declined by 3.8 ms (99.9% CI: -4.1, -3.5) in females and 3.3 ms (99.9% CI: -3.5, -3.1) in males.*”

Lines 87-92 - These should go in the Methods section, that should be after the Introduction.

**We thank the reviewer for this suggestion and agree that the sample analytic counts are more appropriate presented after the Introduction. In revising the manuscript, we removed specific sample size counts from the Introduction and relocated them in the Results section under *Participant Characteristics*, which aligns with the journal’s required manuscript structure (Introduction, Results, Discussion, Materials and Methods;** [**https://journals.plos.org/digitalhealth/s/submission-guidelines**](https://journals.plos.org/digitalhealth/s/submission-guidelines)**).**

**Because PLOS Digital Health places the Methods after the Results, we believe the Results section is the most appropriate location to report the final analytic sample and total number of observation days after filtering. This aligns with our presentation of the flow diagram (lines 107-110): “***The participant flow diagram is presented in* ***Figure 1****. Following eligibility filtering, the analytic sample included 20,968 participants (10,025 female and 10,943 male), contributing 5,109,185 person-days of observation.* ***Table 1*** *presents alcohol use patterns and key cardiovascular and health behavioral metrics by age and biological sex.*”

Line 229 - Add to limitations the recall bias of inputing alcohol consumption manually

**We agree and have revised the Limitations section to explicitly acknowledge recall bias associated with self-reported alcohol intake (lines 230-231): “***First, alcohol intake was self-reported in a real-world setting, which introduces recall bias as well as uncertainty in dose and timing.*”

Line 236 - Add to limitations the effect of the choice of person-mean centering

**We agree and the following to the limitations to acknowledge the implications of this decision (lines 239-242): “***Fifth, we focused on acute physiological and behavioral consequences of alcohol using a within-person (via person-mean centering) framework. Accordingly, findings reflect short-term deviations from an individual’s typical physiology and behavior rather than between-person differences or long-term health implications.*”

Line 258 - Why wasn't the sampling performed after checking for eligibility criteria?

**We appreciate the request for clarification regarding the order of sampling and eligibility screening. Sampling was performed prior to eligibility filtering to reduce computational burden, as querying full multi-million row wearable and journal tables for all active users would have required extracting far more data than were ultimately analyzed. Eligibility criteria where thus applied after sampling. We present the full filtering sequence in Figure 1 for transparency.**

Line 266 - STROBE guidelines suggest to indicate the study design in the title or abstract

**We agree and have revised the Abstract to explicitly state the study design. The opening of the Abstract now specifies that this was a retrospective cohort study, consistent with STROBE reporting recommendations (lines 54-57): “***In this retrospective cohort study, we analyzed 5,109,185 person-days from 20,968 participants and fit generalized additive models to estimate within-person associations between alcohol intake and nocturnal resting heart rate (RHR), heart rate variability (HRV), sleep duration, and next-day physical activity.*”

Line 272 - How were artifacts handled? RMSSD is sensitive to artifacts.

**As addressed in our response to the editor, we have clarified the artifact-handling procedure used in the derivation of nocturnal HRV (lines 284-292): “***RHR and HRV were derived from nocturnal sleep using a weighted average approach, prioritizing more stable periods of slow-wave sleep (28). RHR was defined as the average number of heart beats per minute during the primary sleep episode. HRV was calculated as the root mean squared of successive beat-to-beat interval differences (RMSSD). To mitigate the influence of motion and signal artifacts, epochs identified as wake or as having low signal quality were excluded prior to aggregation. Remaining epochs were combined using a weighted average, with higher weights assigned to epochs with higher likelihood of slow-wave sleep and those occurring later in the sleep period. Sleep duration was defined as total sleep time. WHOOP (WHOOP, Inc. Boston, MA) has been validated against electrocardiography and polysomnography for heart rate (99% agreement) and 2-stage sleep categorization (86-89% agreement) (23, 24).*

Line 278 - How was manual input for physical activity handled with when there was contextual automated detection?

**We thank the reviewer for this question and agree that this distinction warrants clarification. We have revised the Methods to explicitly describe how manual and automated activity entries were reconciled. Manual activity logging is used only to identify activities not automatically detected by the device, whereas activity intensity is derived from continuous heart rate data during these time periods. Participants cannot manually input heart rate zones or intensity. Accordingly, the summated heart-rate-zone score (SHRZS) reflects objectively measured load regardless of whether an activity was detected automatically or logged manually. This clarification has been added to the methods (lines 293-299): “***Daily physical activity was calculated using the summated-heart-rate-zone score (SHRZS), a validated measure of internal training load developed by Edwards (29) and widely used across athletic and occupational populations (30-33). Each participant’s maximum heart rate was estimated using the non-linear formula 192–0·007*age^^2^ (34), manually set, or defined as their highest recorded heart rate. Physical activity was either detected automatically by the WHOOP analytics platform (~85% of cases) or logged manually by the participant to capture activities not automatically detected by the device. Importantly, regardless of detection method, activity intensity was derived from continuous heart rate data rather than manual input.*”

Line 283 - Are there other references about the usage of a composite score?

**Yes, and we have expanded the Methods to include additional references supporting the validity and broader use of the summated heart-rate-zones score. Specifically, we now cite the original development of the methods by Edwards (ref 29), as well as multiple independent studies demonstrating strong associations between this score and markers of internal and external training load (lines 293-304): “***Daily physical activity was calculated using the summated-heart-rate-zone score (SHRZS), a validated measure of internal training load developed by Edwards (29) and widely used across athletic and occupational populations (30-33). Each participant’s maximum heart rate was estimated using the non-linear formula 192–0·007*age^^2^ (34), manually set, or defined as their highest recorded heart rate. Physical activity was either detected automatically by the WHOOP analytics platform (~85% of cases) or logged manually by the participant to capture activities not automatically detected by the device. Importantly, regardless of detection method, activity intensity was derived from continuous heart rate data rather than manual input. Continuous heart rate data during each activity was used to calculate time spent in five zones defined as percentages of maximum heart rate: zone 1 (50-60%), zone 2 (60-70%), zone 3 (70-80%), zone 4 (80-90%), and zone 5 (90-100%). Minutes in each zone were multiplied by zone-specific weights (i.e., zone 1=1, zone 2=2, etc.) and summed to yield the SHRZS. This approach has demonstrated strong associations with session rating of perceived exertion and other internal and external load metrics across diverse populations (30-33)*.”

Line 302 - Why weren't both the moderators included in the model, but modeled separately?

**We appreciate the reviewer’s question. Our moderation analysis were prespecified in the Statistical Analysis Plan and focused on two independent questions:**

1) **Whether deviations in sleep after drinking moderated the effects of alcohol, and**
2) **Whether deviation in physical activity on the days of drinking did the same.**

**Because these questions were defined separately, sleep and activity were modeled as independent moderations in the primary analysis to preserve interpretability and alignment with the preregistered plan.**

**However, in response to reviewer and editor feedback, we conducted additional exploratory models including both moderators simultaneously. In these models, the nonlinear interactions between sleep and activity with drinks remained virtually unchanged in both shape and magnitude, indicating that our findings are robust. We have clarified this rationale in the Statistical Analysis section (lines 348-350): “***Moderators were modeled separately to align with the preregistered analysis plan and to maintain interpretability. Exploratory analysis including both sleep and activity together yielded similar results, supporting the robustness of the primary findings.”*

Line 326 - Why weren't the Bonferroni correction or other methods used, which would decrease the p-value (being 16 models)?

**We understand the reviewers concern that the multiple testing across 16 primary models requires appropriate control of family-wise error. In the revised manuscript, we have taken two complementary approaches. First, we tightened the statical significance threshold to α=0.001 for all sixteen primary models, which is more conservative than a Bonferroni correction of 16 tests (0.05/16=0.003). Second, all pairwise comparisons within each model were conducted using a multivariate t-distribution, which controls the family-wise error rate within each model family. These changes have been described in greater detail above in our second response to the Editor, as well as in the Statistical Analysis section (lines 341-343; 353-355).**

Table 1 - Are the numbers after ± standard deviations? How can most of the comparisons be statistically significant when the standard deviations are so high?

**Thank you for this astute observation. In revisiting Table 1, we identified that several variables were not normally distributed. Normality was assessed using the Shapiro-Wilk test and inspection of Q-Q plots, and summary statistics were revised accordingly. Table 1 now reports normally distributed variables as mean ± standard deviation and non-normally distributed variables as median (interquartile range). The table legend has been updated to clearly indicate this distinction. Regarding the high number of statistically significant comparisons, the large sample size (n=20,968) increases statistical power, making even small group differences statistically significant.**

Tables S1-S14: All the p-values reported are hypothesis tested. They should be factored into the correction for multiple comparisons

**We appreciate this concern and agree that multiple comparison control must be explicit. We have clarified in the revised Statistical Analysis section.**

**Specifically, all primary models were conducted using a conservative significance threshold (α=0.001) across the sixteen primary models, which is more stringent than a Bonferroni correction for 16 tests (0.05/16 = 0.003). In addition, all pairwise contrasts reported in Tables S1-S14 were evaluated using a multivariate t-distribution, which controls family wise error rate within each model family.**

**To further contextualize inference beyond p-values, we have also added standardized marginal effect sizes (ES) for all contrasts in the Supplementary tables.**
